# Low expression of NR1H3 correlates with macrophage infiltration and indicates worse survival in breast cancer

**DOI:** 10.3389/fgene.2022.1067826

**Published:** 2023-01-09

**Authors:** Jing Zhang, Jiawen Zhang, Weiwei Zhao, Qingxian Li, Wenwu Cheng

**Affiliations:** ^1^ Department of Integrated Therapy, Shanghai Cancer Center, Fudan University, Shanghai, China; ^2^ Department of Oncology, Shanghai Medical College, Fudan University, Shanghai, China; ^3^ Department of Obstetrics and Gynecology, Shanghai General Hospital, Shanghai Jiao Tong University School of Medicine, Shanghai, China; ^4^ The Center of Reproductive Medicine, Second Affiliated Hospital of Naval Medical University, Shanghai, China

**Keywords:** NR1H3, immune infiltrates, macrophages, prognosis, breast cancer

## Abstract

**Background:** Nuclear receptor NR1H3 is a key regulator of macrophage function and lipid homeostasis. Here, we aimed to visualize the prognostic value and immunological characterization of NR1H3 in breast cancer.

**Methods:** The expression pattern and prognostic value of NR1H3 were analyzed via multiple databases, including TIMER2, GEPIA2 and Kaplan-Meier Plotter. TISIDB, TIMER2 and immunohistochemical analysis were used to investigate the correlation between NR1H3 expression and immune infiltration. GO enrichment analysis, KEGG analysis, Reactome analysis, ConsensusPathDB and GeneMANIA were used to visualize the functional enrichment of NR1H3 and signaling pathways related to NR1H3.

**Results:** We demonstrated that the expression of NR1H3 was significantly lower in breast cancer compared with adjacent normal tissues. Kaplan-Meier survival curves showed shorter overall survival in basal breast cancer patients with low NR1H3 expression, and poorer prognosis of relapse-free survival in breast cancer patients with low NR1H3 expression. NR1H3 was mainly expressed in immune cells, and its expression was closely related with infiltrating levels of tumor-infiltrating immune cells in breast cancer. Additionally, univariate and multivariate analysis indicated that the expression of NR1H3 and the level of macrophage infiltration were independent prognostic factors for breast cancer. Gene interaction network analysis showed the function of NR1H3 involved in regulating of innate immune response and macrophage activation. Moreover, NR1H3 may function as a predictor of chemoresponsiveness in breast cancer.

**Conclusion:** These findings suggest that NR1H3 serves as a prognostic biomarker and contributes to the regulation of macrophage activation in breast cancer.

## Introduction

Breast cancer is the most common malignancy and the second leading cause of cancer related-deaths among women worldwide (Bray et al., 2018). Despite advancements in treatment regimens, the mortality of breast cancer remains a challenge (Gradishar et al., 2015; Biglia et al., 2016). The 5-year overall survival rate of breast cancer patients with distant metastasis is only approximately 25% (Coleman et al., 2008). The subtypes of breast cancer are based on the expressions of estrogen receptor (ER), progesterone receptor (PR) and human epidermal growth factor 2 (HER-2) (Payne et al., 2008; Rakha et al., 2010). Although the hormone receptors, tumor size/grade and number of axillary node metastases have been widely used as prognostic biomarkers in the case of breast cancer, these factors are limited to predict patient’s survival with specific subtypes. There is a need to identify reliable biomarkers to predict the clinical outcome of breast cancer regardless of tumor heterogeneity effectively.

Macrophages are an important part of infiltrating immune cells in tumor microenvironment (TME).They are always abundantly present in breast cancer (Pollard, 2008). In the last decade, accumulating evidences have revealed that macrophages can participate in tumorigenesis by mediating immune escape, metastasis and tumor angiogenesis (Li X. et al., 2017; De Palma et al., 2017; Cully, 2018; Zhu et al., 2019). Nuclear receptor NR1H3 is a key regulator of macrophage function and lipid homeostasis (Joseph et al., 2004; Mitro et al., 2007; Bensinger et al., 2008; Fessler, 2008; Zelcer et al., 2009), especially playing a central role in the anti-inflammatory response in macrophages (Duc et al., 2019). The low expression of NR1H3 is a poor prognostic factor for muscle‐invasive bladder cancer (Wu et al., 2017). In breast cancer, recent studies showed that NR1H3 is likely to be an onco-suppressor gene and related to immune infiltration (Vedin et al., 2009; Garattini et al., 2016; Yu et al., 2021). However, the prognostic value and immunological characterization of immune-related gene NR1H3 in breast cancer remain unclear.

In this study, we visualized the prognostic landscape of NR1H3 in breast cancer using databases, including TIMER2, GEPIA2, and Kaplan-Meier Plotter. We also explored the potential relationship between NR1H3 expression and macrophage infiltration level using the TIMER2 and TISIDB databases. Our results indicate that NR1H3 influences the prognosis of patients with breast cancer, probably *via* its interaction with infiltrating macrophages. Immune-related gene NR1H3 is likely to be one of potential immune markers for breast cancer immunotherapy.

## Materials and methods

### NR1H3 expression analysis

TIMER2 database (http://timer.cistrome.org/) was used to show the expression difference of NR1H3 between tumor and adjacent normal tissues in different cancer types of the TCGA project (Li et al., 2020). The “Expression analysis-Box Plots” module of the GEPIA2 web server (http://gepia2.cancer-pku.cn/#analysis) was used to obtain box plot of the expression difference between the breast tumor tissues and the corresponding normal tissues of the GTEx database (Tang et al., 2019). Additionally, the NR1H3 expressions in different pathologicals and clinical stages were obtained using the UALCAN database (http://ualcan.path.uab.edu/analysis-prot.html) (Chandrashekar et al., 2017). The Oncomine database (http://www.oncomine.org) was used to validate the expression of NR1H3 in breast cancer (Rhodes et al., 2007).

### Human tissue microarray and immunohistochemical analysis

Paired human breast cancer and adjacent non-tumor paraffin tissue microarrays were purchased from Shanghai Zuocheng Biotech (Shanghai, China). The sections were subjected to antigen retrieval and incubated with primary antibodies against NR1H3 (ab41902, abcam) and CD68 (ab955, abcam) at 4°C in a humid chamber overnight. The next day, the sections were incubated with biotinylated secondary antibody for 60 min. Protein levels of NR1H3 and CD68 were evaluated as follows: the slides were appraised for the intensity of the staining (0–3) and the percentage of positively stained cells (0–4). Index of protein levels was calculated as the intensity of the staining × the percentage of positively stained cells. Therefore, slices were divided into 4 groups: negative (score 0), low expression (score 1–4), medium expression (score 5–8) and high expression group (score 9–12).

### Subtypes of breast cancer

The subtypes of breast cancer for sub-group analysis are divided based on the 2013 StGallen criteria using the expression of HER2, ESR1 and MKI67, including basal (ESR1-/HER2-), luminal A (ESR1+/HER2-/MKI67 low), luminal B (ESR1+/HER2+/MKI67 high) and HER2(HER2+/ESR1-).

### Survival analysis

GEPIA2 and GSCA (http://bioinfo.life.hust.edu.cn/GSCA/#/) databases were used to reveal the correlation between NR1H3 expression and overall survival (OS), disease-free survival (DFS) or progress free survival (PFS) of breast cancer patients (Tang et al., 2019). Kaplan-Meier Plotter (https://kmplot.com/analysis/) was used to assess the effect of NR1H3 on OS, relapse-free survival (RFS), distant metastasis-free survival (DMFS) and post-progression survival (PPS) in breast cancer (Lanczky et al., 2016). Hazard ratios (HRs) with 95% confidence intervals (CI) and log-rank *p*-values were calculated. Additionally, we constructed univariate and multivariate Cox proportional hazard models. Multivariate analysis comprised seven variables, including the expression of NR1H3 gene, macrophage level, age, tumor stage, gender, race and tumor purity. The survival curves, featuring patterns of NR1H3 gene expression and macrophage level were shown on the diagram. The association between each immune cell type and OS was displayed under the low or high expression of NR1H3.

### Immune infiltration analysis

The correlation between NR1H3 expression and immune infiltration was determined using the TISIDB, TIMER and TIMER2. TISIDB (http://cis.hku.hk/TISIDB/index.php) was used to show the relations between NR1H3 expression and abundance of 28 tumor-infiltrating lymphocytes (TILs) types, immunoinhibitors, immunostimulators, MHC molecules, chemokines and chemokine receptors (Li T. et al., 2017; Ru et al., 2019). The TIMER2 online tool (http://timer.cistrome.org/) was used to analyze the correlation of NR1H3 with the infiltration level and prognostic value of immune cells, including macrophages, CD4^+^ T Cells, CD8^+^ T Cells, monocytes, B Cells, dendritic cells (DCs), neutrophils and natural killer (NK) cells (Li et al., 2020). We also used the TIMER2 to explore the immune infiltration distribution between different somatic copy number changes of NR1H3, and analyze the correlation between the expression of NR1H3 with monocyte markers (CD86, CD115/CSF1R, CD14), macrophage markers (CCL2, CD68, IL10, CD80), M1 macrophage markers (IRF5, INOS/NOS2, COX2/PTGS2), M2 macrophage markers (CD163, VSIG4, MS4A4A) and immune checkpoint molecules (PD-1/CD274, PD-L1/PDCD1, PD-L2/PDCD1LG2 and CTLA-4).

### Single-cell analysis

The scRNA-seq database TISCH (http://tisch.comp-genomics.org) was used to show the detailed cell-type annotation at the single-cell level in breast cancer (Sun et al., 2021). Sub-expression analysis of GEPIA 2021 (http://gepia2021.cancer-pku.cn/) visualized the NR1H3 expression in each immune cell type (B Cells, CD4^+^ T Cells, CD8^+^ T Cells, NK cells and macrophages) available in TCGA/GTEx sub-datasets.

### Genes mutation prediction analysis

The muTarget database (http://www.mutarget.com) is a cancer biomarker/target discovery tool that can identify mutations resulting in expression change. We used the database to predict the mutant genes that affect the expression of NR1H3 gene.

### Interaction network and functional enrichment analysis

The gene ontology (GO) term enrichment analysis was performed by the LinkedOmics database (http://www.linkedomics.org/) pathway analysis. Gene Set Enrichment Analysis (GSEA) was used to search for Kyoto Encyclopedia of Genes and Genomes (KEGG) and Reactome pathways enrichment analysis. The network neighborhoods of NR1H3 were visualized by ConsensusPathDB-human (http://consensuspathdb.org). These data originate from currently 32 public resources for interactions (Kamburov et al., 2009). The GeneMANIA (http://genemania.org/), an online tool for investigation into associated or similar genes for target genes, was used to validate the gene interaction network results and conduct functional enrichment analysis (Franz et al., 2018).

### Receiver operating characteristics plotter

The ROC Plotter platform (http://www.rocplot.org/) was used to identify NR1H3 whether predicts benefit from endocrine therapy and chemotherapy (Fekete and Győrffy, 2019). The platform integrates multiple gene expression datasets at transcriptome level and contains 3,104 breast cancer patients with treatment and response data. The ROC Plotter is a validation tool for predictive biomarkers.

### Statistical analysis

The Kaplan‐Meier plotter, GSCA and GEPIA2 databases were used for generating survival plots, with data including either HR and *p*‐values or *p*‐values derived from a log‐rank test. The Cox proportional hazards regression model was used for univariate and multivariate analyses to evaluate the independence of NR1H3 in predicting prognosis. The correlation of gene expression was assessed by Spearman’s correlation analysis. *p*-values <0.05 were considered as statistically significant.

## Results

### Assessment of NR1H3 expression in breast cancer

**FIGURE 1:**
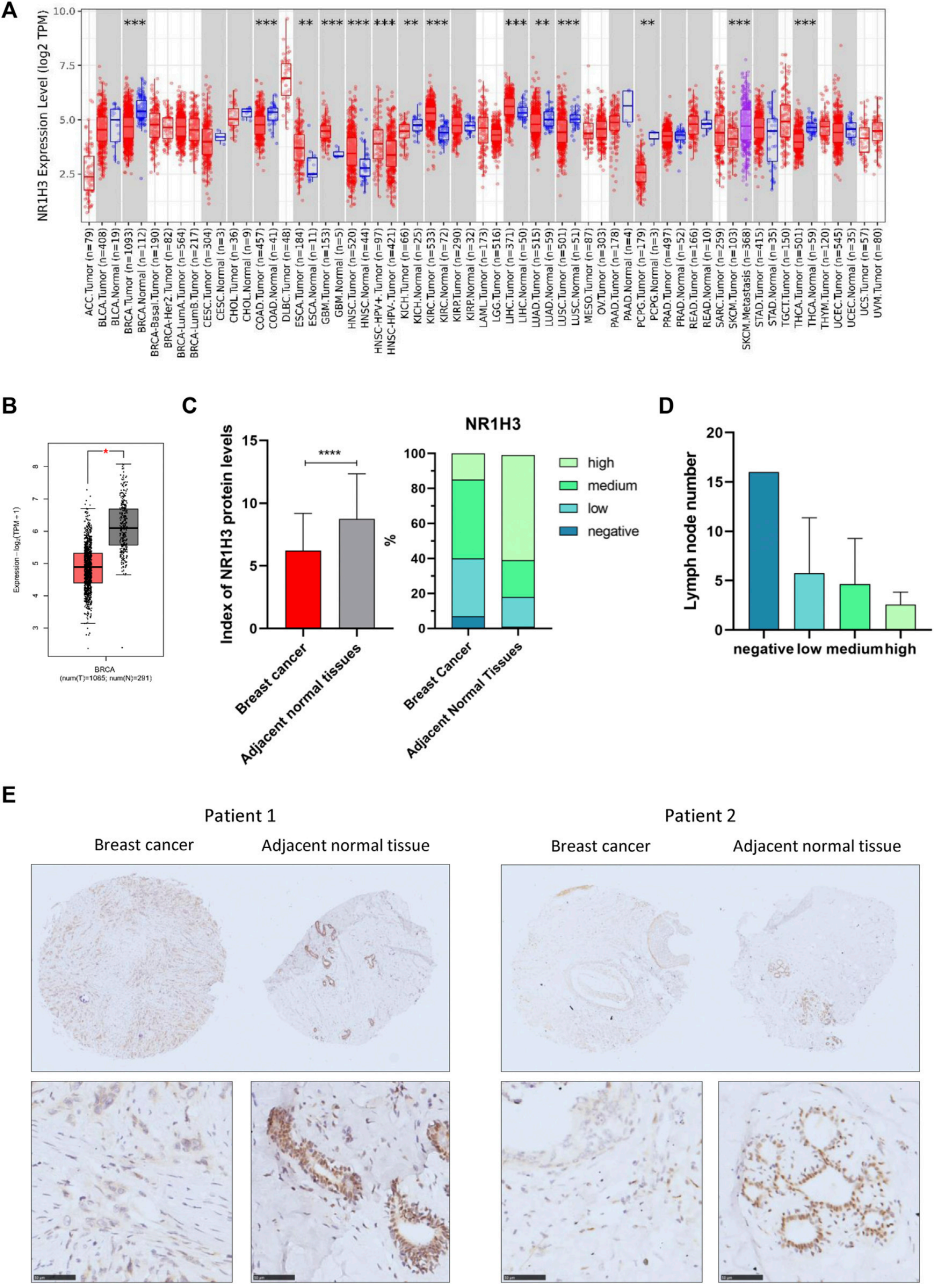
NR1H3 expression in different types of human cancers. **(A)** High or low expression of NR1H3 in different human cancer tissues compared with normal tissues from the TCGA database in TIMER2. **(B)** The level of NR1H3 expression in BRCA using GEPIA2 database. **(C)** Index of NR1H3 protein expression and IHC score groups distribution in breast cancer and adjacent normal tissues. p < 0.0001 **(D)** The number of lymph node metastasis in different NR1H3 protein expression groups in breast cancer. **(E)** Immunohistochemistry analysis of NR1H3 protein in breast cancer and adjacent normal tissues. Scale bar = 50 μm. BRCA, breast invasive carcinoma; IHC, immunohistochemical. *p < 0.05, **p < 0.01, ***p < 0.001, ****p < 0.0001.

To determine the expression pattern of NR1H3 in breast cancer, we analyzed the NR1H3 expression profile based on multiple public databases. As shown in Figures 1A,B, expression of NR1H3 was significantly lower in breast invasive carcinoma (BRCA) compared with adjacent normal tissues. Three breast datasets in the Oncomine were adopted for the validation of lower NR1H3 expression in breast cancer (Supplementary Figure S1A–C). Immunohistochemistry analysis using the tissue microarray (including 83 paired breast cancer and adjacent normal breast tissues) showed that the protein level of NR1H3 was significantly downregulated in breast cancer compared to adjacent normal tissues (Figures 1C,E, *p* < 0.0001). In addition, according to the clinical data of these cancer cases, we found that the lower expression of NR1H3, the more lymph node metastasis (Figure 1D, *p* > 0.05). Then we analyzed the expression of NR1H3 in BRCA based on tumor subclasses using the UALCAN database. Luminal (*p* < 1.0e-12), HER2Pos (*p* = 4.1e-10), TNBC Basal-like 1 (TNBC-BL1) (*p* = 5.2e-03), TNBC Basal-like 2 (TNBC-BL2) (*p* = 1.54e-5), TNBC mesenchymal stem-like (TNBC-MSL) (*p* = 6.26e-5), TNBC Mesenchymal (TNBC-M) (*p* = 4.4e-16), TNBC unspecified (TNBC-UNS) (*p* = 1.0e-4) showed lower NR1H3 expression compared with normal tissues (Supplementary Figure S1D). No statistical difference was found in NR1H3 mRNA expression among different tumor stages (*p* > 0.05) (Supplementary Figure S1E). Moreover, the expression of NR1H3 was also significantly lower in other types of cancers compared with the corresponding normal tissues, such as colon adenocarcinoma (COAD), kidney chromophobe (KICH), lung adenocarcinoma (LUAD) and thyroid carcinoma (THCA) tissues (Figure 1A).

### Correlation of NR1H3 with survival in different subtype of breast cancer

**FIGURE 2:**
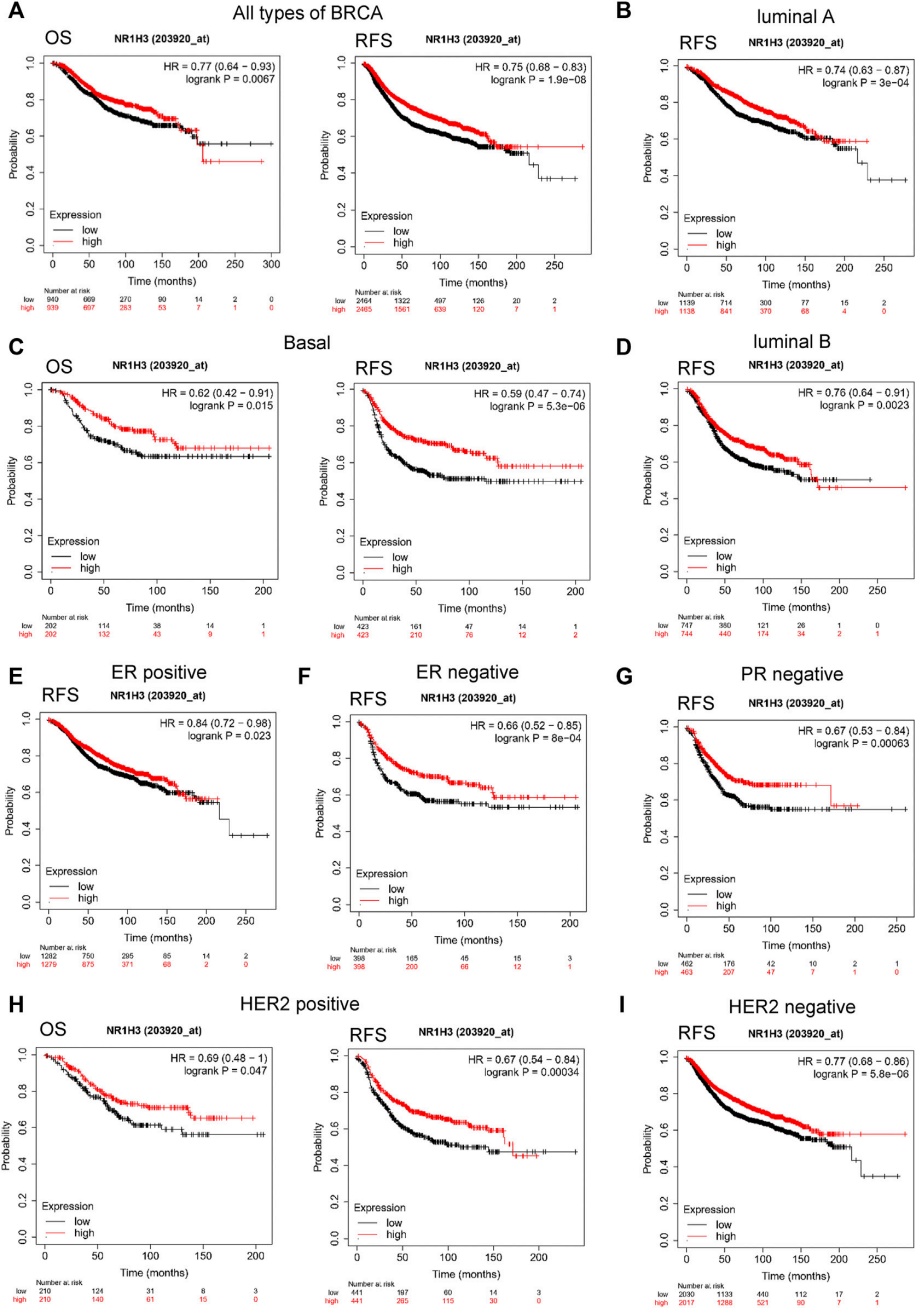
Kaplan-Meier survival curves of OS and RFS comparing the high and low expression of NR1H3 in breast cancer. **(A)** In the Kaplan-Meier plotter database, low expression of NR1H3 indicated a worse survival prognosis of OS, RFS in breast cancer patients. **(B)** RFS survival curve of luminal A breast cancer patients. **(C)** OS and RFS survival curve of basal breast cancer patients. **(D)** RFS survival curve of luminal B breast cancer patients. **(E)** RFS survival curve of ER-positive breast cancer patients. **(F)** RFS survival curve of ER-negative breast cancer patients. **(G)** RFS survival curve of PR-negative breast cancer patients. **(H)** OS and RFS survival curves of HER2-positive breast cancer patients. **(I)** RFS survival curve of HER2-negative breast cancer patients. OS, overall survival; RFS, relapse-free survival; ER, estrogen receptor; PR, progesterone receptor; HER-2, human epidermal growth factor 2.

To evaluate the value of NR1H3 in predicting the prognosis of breast cancer patients, the association between NR1H3 expression and clinical prognosis of OS, DFS and RFS was analyzed in TCGA cohort. Kaplan-Meier survival curves showed that OS was shorter in BRCA patients with low NR1H3 expression in the GEPIA2 and GSCA databases (Supplementary Figure 2A,B). Then we used the Kaplan-Meier plotter approach to conduct a group of survival analyses using gene probe 203920_at. Similarly, the poor prognosis in breast cancer (OS *p* = 0.007; RFS *p* = 1.9e-8; DMFS *p* = 0.004; PPS *p* = 0.011) was shown to correlate with lower NR1H3 expression (Figure 2A; Supplementary Figure S2C). It is well known that breast cancer is a heterogeneous tumor and is divided into different subtypes based on ER/PR and HER-2 expression (Colombo et al., 2011). As shown in Figure 2; Supplementary Figure 2, OS (*p* = 0.015), DMFS (*p* = 0.004) and PPS (*p* = 0.023) were shorter in basal breast cancer patients with low NR1H3 expression, but not in luminal A, luminal B and HER2+ breast cancer patients (*p* > 0.05). Moreover, low NR1H3 expression was correlated with poorer prognosis of RFS in basal (*p* = 5.3e-6), luminal A (*p* = 3.0e-4), luminal B (*p* = 0.002) and HER2+ breast cancer patients (*p* = 0.0003) (Figure 2).

We further explored the prognostic characteristics of NR1H3 under different ER, PR, and HER-2 status. ER-positive subtype had shorter RFS (*p* = 0.023) in breast cancer with low NR1H3 expression (Figure 2E). Low NR1H3 expression was only correlated with worse PPS in PR-positive subtype (*p* = 0.03) (Supplementary Figure S2D). ER-negative (RFS p = 8e-04; DMFS *p* = 0.006) and PR-negative (RFS *p* = 6.3e-04; DMFS *p* = 0.045) subtypes were also statistically associated with clinical prognosis of RFS and DMFS, but only a trend towards poor survival without statistical significance of OS and PPS in NR1H3-low breast cancer (Figures 2F,G, Supplementary Figure S2F,G). Compared with high NR1H3 expression, low expression of NR1H3 indicated a worse survival prognosis of OS (*p* = 0.047), RFS (p = 3e-04) and PPS (*p* = 0.033) in HER2-positive breast cancer (Figure 2H; Supplementary Figure S2H). Among HER2-negative, only RFS (*p* = 5.8e-06) and DMFS (*p* = 0.023) showed statistical survival differences (Figure 2I and Supplementary Figure S2I). In addition, the correlation of NR1H3 expression with clinical and pathological features from Kaplan-Meier Plotter was integrated in Table 1. For instance, RFS was shorter in lymph node positive breast cancer patients with low NR1H3 expression (*p* = 0.015), but not in lymph node negative breast cancer patients (*p* = 0.058). For grade 3 breast cancer patients, low expression of NR1H3 indicated a worse survival prognosis of OS (*p* = 0.01), RFS (*p* = 0.025) and DMFS (*p* = 0.035). These results suggest that low NR1H3 expression may be a risk factor for a poor prognosis in breast cancer patients.

**TABLE 1:** Correlation of NR1H3 gene expression with OS, RFS, DMFS and PPS in breast cancer with different clinicopathological features.

Clinicopathological characteristics	Overall survival			Relapse-free survival			Distant metastasis-free survival			Post-progression survival		
	*N*	Hazard ratio	*p-*value	*n*	Hazard ratio	*p-*value	*n*	Hazard ratio	*p-*value	*n*	Hazard ratio	*p-*value
Intrinsic subtype												
basal	404	0.62 (0.42–0.91)	**0.015**	846	0.59 (0.47–0.74)	**5.3e-06**	571	0.63 (0.46–0.87)	**0.004**	76	0.52 (0.3–0.92)	**0.023**
luminal A	794	0.83 (0.6–1.14)	0.245	2277	0.74 (0.63–0.87)	**3e-04**	1260	0.87 (0.67–0.13)	0.301	204	1.04 (0.73–1.49)	0.831
luminal B	515	0.73 (0.52–1.04)	0.081	1491	0.76 (0.64–0.91)	**0.002**	756	0.76 (0.57–1)	0.051	139	0.7 (0.46–1.07)	0.101
HER2 positive	166	0.8 (0.45–1.41)	0.444	315	0.82 (0.58–1.17)	0.269	178	1.03 (0.63–1.7)	0.894	39	0.52 (0.25–1.11)	0.087
ER status - IHC and array												
positive	720	0.86 (0.62–1.2)	0.379	2561	0.84 (0.72–0.98)	**0.023**	1109	0.93 (0.71–1.22)	0.599	195	0.78 (0.54–1.14)	0.200
negative	349	0.77 (0.52–1.14)	0.185	796	0.66 (0.52–0.85)	**8e-04**	518	0.65 (0.47–0.89)	**0.006**	66	0.6 (0.32–1.11)	0.101
PR status - IHC												
positive	156	0.46 (0.21–1.02)	0.052	926	0.87 (0.65–1.16)	0.343	529	1.1 (0.7–1.73)	0.675	32	0.31 (0.11–0.94)	**0.030**
negative	291	0.66 (0.4–1.08)	0.098	925	0.67 (0.53–0.84)	**6e-04**	637	0.74 (0.56–0.99)	**0.045**	37	1.81 (0.67–4.89)	0.239
HER2 status - array												
positive	420	0.69 (0.48–1)	**0.047**	882	0.67 (0.54–0.84)	**3e-04**	451	0.81 (0.58–1.12)	0.203	822	0.61 (0.39–0.97)	**0.033**
negative	1459	0.81 (0.65–1.01)	0.059	4047	0.77 (0.68–0.86)	**5.8e-06**	2314	0.82 (0.69–0.97)	**0.023**	347	0.78 (0.6–1.02)	0.073
Lymph node status												
positive	452	0.72 (0.52–1.01)	0.054	1656	0.81 (0.68–0.96)	**0.015**	889	0.94 (0.73–1.2)	0.615	153	0.81 (0.54–1.22)	0.3060
negative	726	0.77 (0.55–1.09)	0.143	2368	0.86 (0.73–1.01)	0.058	1309	0.82 (0.64–1.05)	0.110	184	0.75 (0.5–1.13)	0.166
Grade												
1	175	0.97 (0.41–2.3)	0.941	397	1.12 (0.67–1.86)	0.668	239	1.33 (0.59–3)	0.490	35	0.65 (0.24–1.8)	0.490
2	443	0.79 (0.53–1.19)	0.264	1177	0.81 (0.65–1)	0.053	798	0.87 (0.65–1.16)	0.334	142	0.8 (0.51–1.27)	0.341
3	586	0.67 (0.5–0.91)	**0.010**	1300	0.81 (0.67–0.97)	**0.025**	836	0.76 (0.58–0.98)	**0.035**	187	0.71 (0.5–1.02)	0.063

OS, overall survival; RFS, relapse-free survival; DMFS, distant metastasis-free survival; PPS, post progression survival; ER, estrogen receptor; PR, progesterone receptor; HER-2, human epidermal growth factor 2.

*p*-values < 0.05 are displayed in bold.

### Correlation analysis between NR1H3 expression and infiltrating immune cells

**FIGURE 3:**
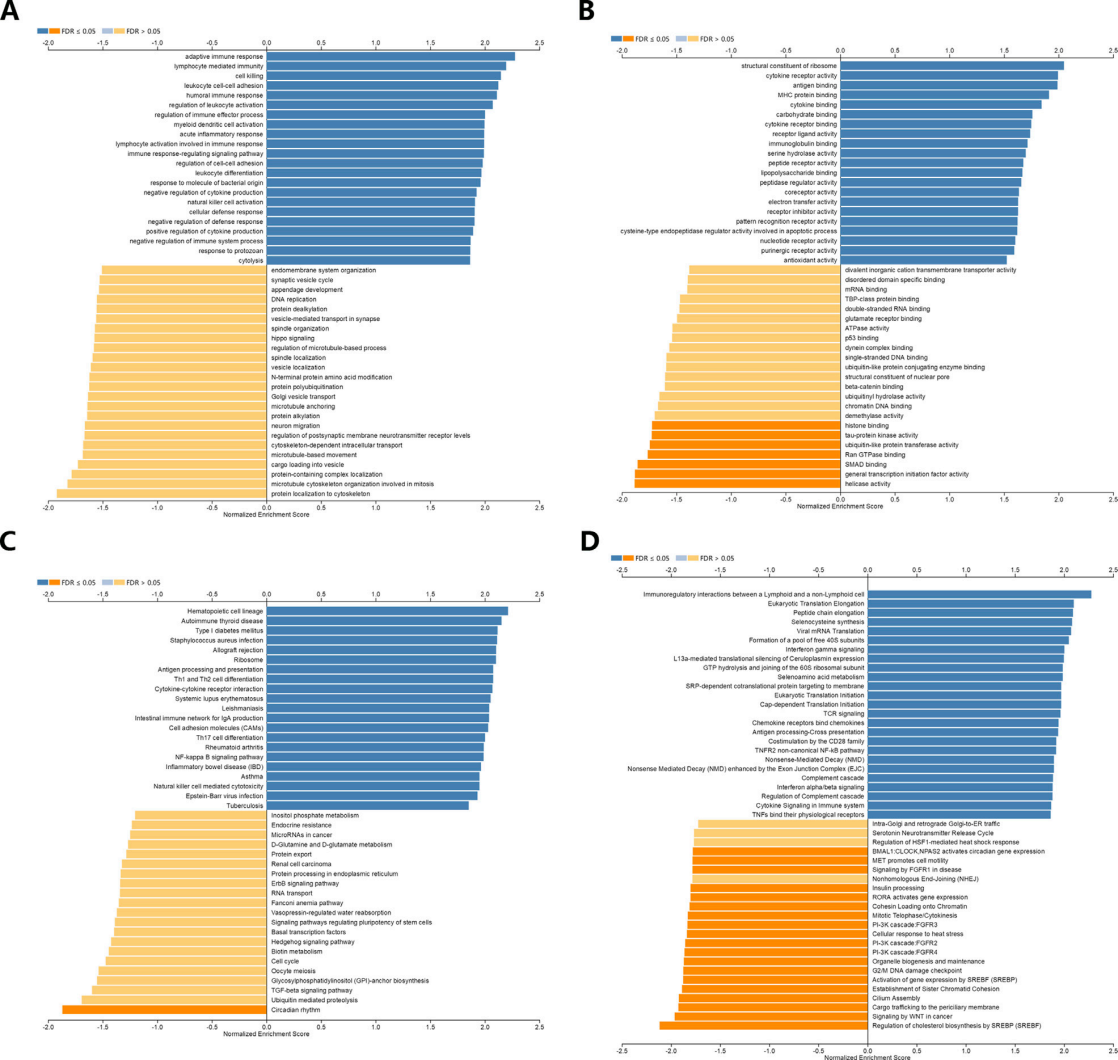
Function and pathway enrichment analyses of NR1H3 in breast cancer. **(A,B)** Significant Gene Ontology terms of NR1H3, including biological processes (BP) and molecular function (MF). **(C,D)** Significant GSEA results of NR1H3, including KEGG pathways and Reactome pathways. GSEA, Gene Set Enrichment Analysis; KEGG, Kyoto Encyclopedia of Genes and Genomes.

**FIGURE 4:**
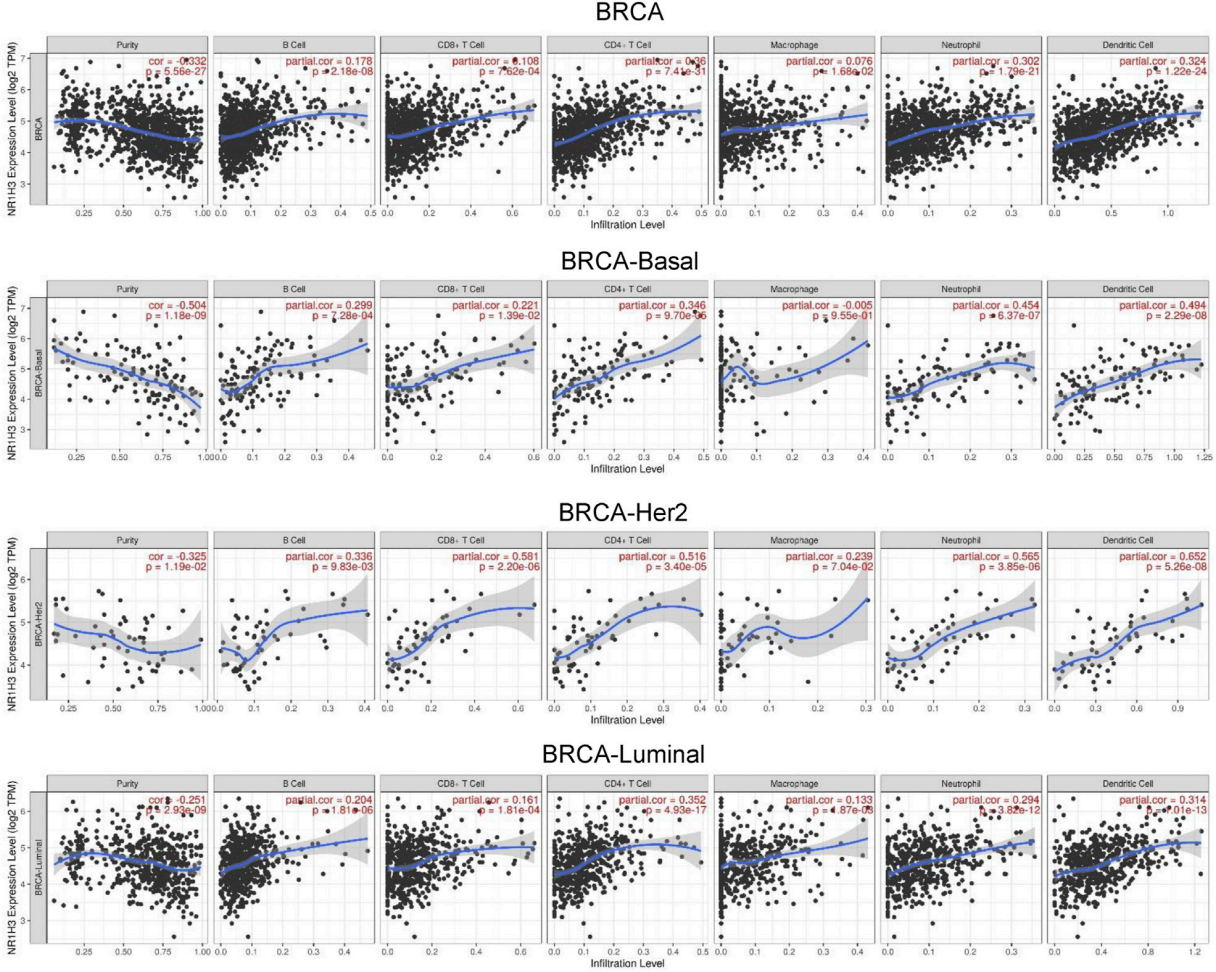
Correlation of NR1H3 expression with 6 types of infiltrating immune cells (B Cells, CD4^+^ T Cells, CD8^+^ T Cells, neutrophils, macrophages, and DCs) in BRCA and each subtype available in TIMER. DCs, dendritic cells; BRCA, breast invasive carcinoma.

GO enrichment analysis revealed adaptive immune response and immune cells activation process were correlated with the expression of NR1H3 in breast cancer (Figures 3A,B). Additionally, the signaling pathways were significantly enriched of NR1H3 by KEGG analysis and Reactome analysis were presented in Figures 3C,D. Tumor-infiltrating immune cells, as prominent components of the TME, are closely linked to the initiation, progression or metastasis of cancer (Fridman et al., 2011; Steven and Seliger, 2018). Here, we found the relations between abundance of 28 TIL types and expression of NR1H3 were strongly correlated across different human cancer types (Supplementary Figure S3). Specifically, NR1H3 expression was closely related with infiltrating levels of TIL in BRCA. Next, we analyzed the correlation between NR1H3 expression and 6 types of infiltrating immune cells (B Cells, CD4^+^ T Cells, CD8^+^ T Cells, neutrophils, macrophages and DCs) in BRCA using TIMER database. Consistently, Figure 4 showed that NR1H3 expression level had significantly positive correlations with infiltrating levels of B Cells (r = 0.178, *p* = 2.18e-8), CD8^+^ T Cells (r = 0.108, *p* = 7.62e-4), CD4^+^ T Cells (r = 0.36, *p* = 7.41e-31), macrophages (r = 0.076, *p* = 1.68e-2), neutrophils (r = 0.302, *p* = 1.79e-21), and DCs (r = 0.324, *p* = 1.22e-24) in BRCA and with negative correlation with tumor purity (r = -0.332, *p* = 5.56e-27). Moreover, the same trend results were found in each subtype (Figure 4). These findings strongly suggest that NR1H3 is correlated with immune cells infiltration in breast cancer.

Next, TIMER, CIBERSORT, CIBERSORT-ABS, QUANTISEQ, XCELL, MCPCOUNTER and EPIC algorithms were further used to validate the potential relation between the expression of NR1H3 and the infiltration level of 8 types of immune cells (B Cells, CD4^+^ T Cells, CD8^+^ T Cells, monocytes, macrophages, DCs, neutrophils and NK cells) in diverse cancer types of TCGA. As shown in Supplementary Figure S4, CD8^+^ T Cells and macrophages were two immune cell types most strongly correlated with NR1H3 expression in BRCA.

### Association between NR1H3 copy number variations and immune infiltrates

**FIGURE 5:**
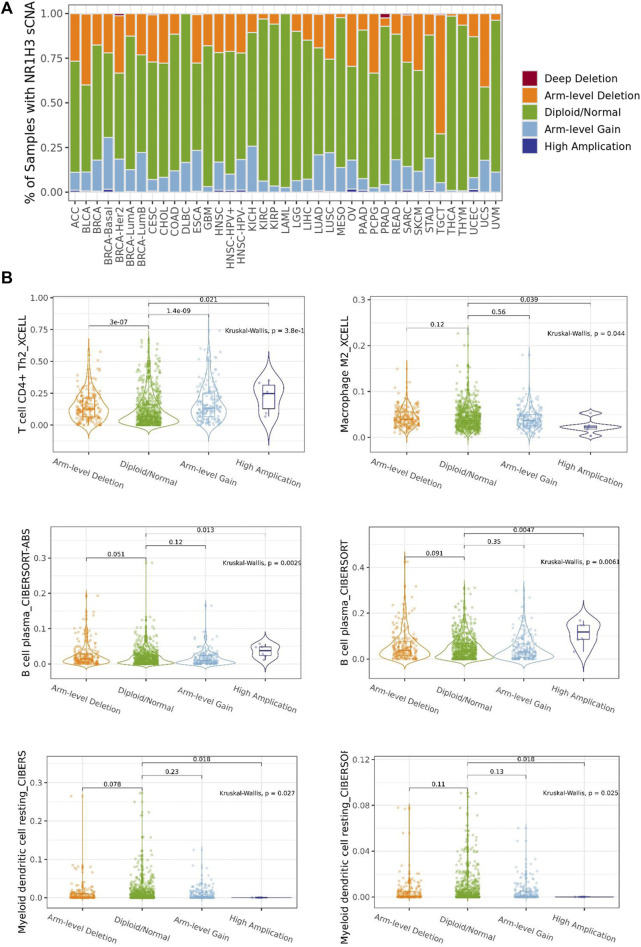
The association between NR1H3 copy number variations and immune infiltrates. **(A)** A stacked bar plot showed the relative proportion of different sCNA states of the NR1H3 for all TCGA cancer types. **(B)** Analysis according to different groups of sCNA showed a significant difference in NR1H3 expression at the CD4^+^ Th2 cell, M2 macrophage, B Cell and DC cell levels among these groups in BRCA. sCNA, somatic copy number alterations. DC, dendritic cell. *p < 0.05, **p < 0.01, ***p < 0.001.

The association between NR1H3 copy number variations (including deep deletion, arm-level deletion, diploid/normal, arm-level gain and amplification) and immune infiltrates in BRCA was investigated using different algorithms of TIMER2. The immune infiltration distribution by the somatic copy number alterations (sCNA) status of NR1H3 across TCGA cancer types was demonstrated in Figure 5A. Then, six of significant relationships between the changes in NR1H3 copy number variations and immune infiltrates in BRCA using TIMER2 were presented (Figure 5B). In particular, arm-level deletion (p = 3e-07), arm-level gain (*p* = 1.4e-09) and high amplification (*p* = 0.021) of NR1H3 had significant correlation with CD4^+^ Th2 cell infiltration level using XCELL algorithms. High amplification of NR1H3 was associated with low M2 macrophage infiltration level, compare with the “diploid/normal” status (*p* = 0.039). By CIBERSORT and CIBERSORT-ABS algorithms, high amplification of NR1H3 had high B Cell and low DC cell infiltration (Figure 5B). However, no statistical difference was found in CD8^+^ T Cell, neutrophil, and NK cell infiltration from TIMER2 (data not shown). These findings indicate the potential mechanism by which NR1H3 alterations affect immune infiltration distribution.

### Correlation between NR1H3 expression and tumor infiltrating macrophages in BRCA

**FIGURE 6:**
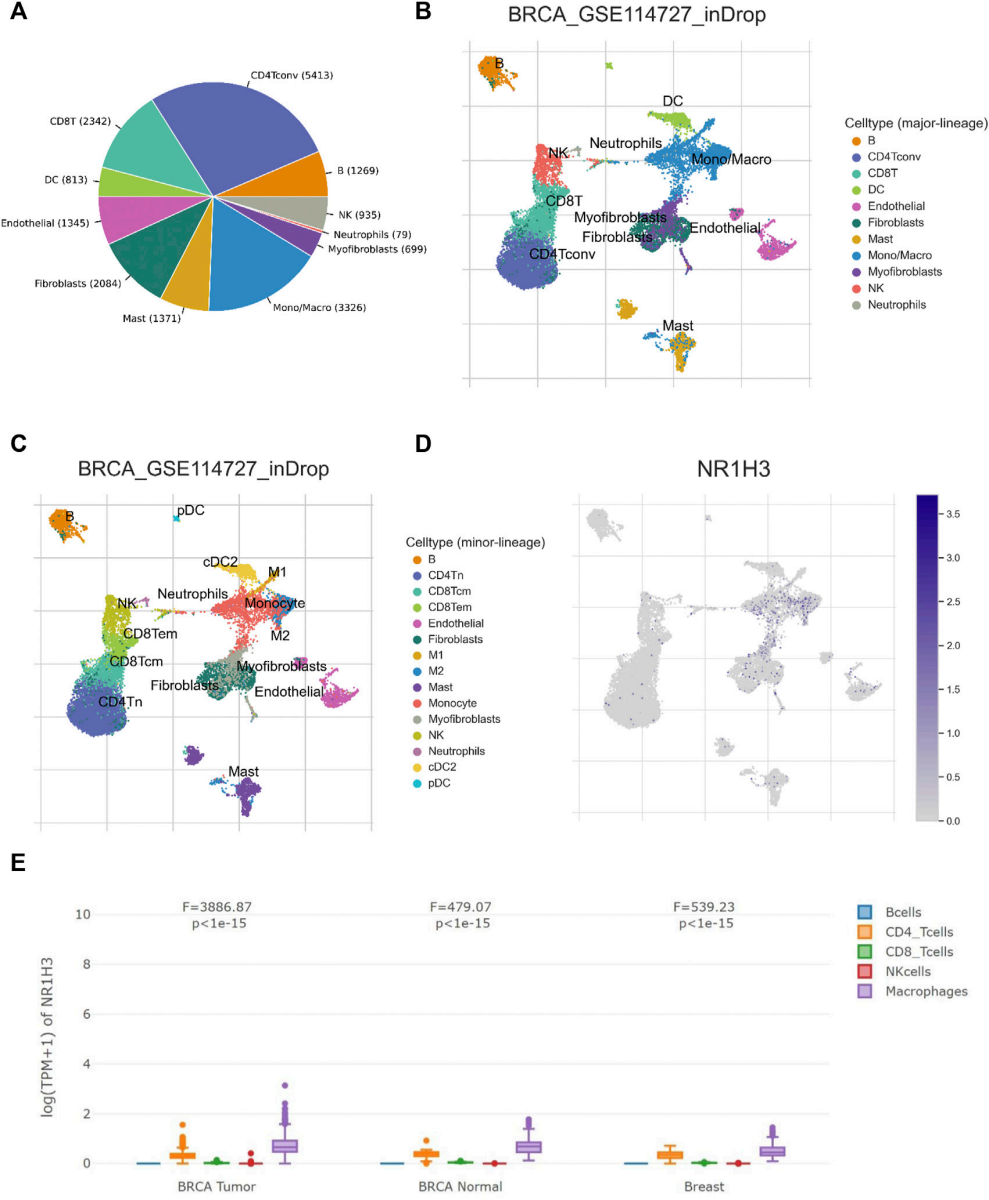
NR1H3 expression in TME-related cells. **(A–C)** The TME cell types and distribution in the GSE114727_inDrop dataset. **(D)** The distribution of NR1H3 in different cell types was analyzed using single-cell resolution in the GSE114727_inDrop dataset using the TISCH database. **(E)** Comparison of NR1H3 expression distribution across samples in BRCA Tumor/BRCA normal from TCGA and breast tissue from GTEx. TME, tumor microenvironment; TISCH, Tumor Immune Single Cell Hub; BRCA, breast invasive carcinoma.

Considering the role of NR1H3 in immune infiltrates in BRCA and its prognostic impact, we used six BRCA data sets (BRCA_SRP114962, BRCA_GSE143423, BRCA_GSE138536, BRCA_GSE136206_mouse_aPD1aCTLA4, GSE114727-inDrop, BRCA_GSE114727_10X and BRCA_GSE110686) in the TISCH platform to analyze the expression of NR1H3 at the single-cell level. The results showed higher NR1H3 expressions in immune cells, mainly in monocyte/macrophage, compared with malignant cells (Supplementary Figure S5). Then we analyzed the GSE114727-inDrop dataset, which is divided into 12 types of cells. Figures 5A–D showed the number of cells in each cell type, with the distribution and number of various TME-related cells presented. In this data set, CD4^+^ T Cells were the most abundant immune cells (n = 5,413), whereas NR1H3 was highly expressed in monocyte/macrophage (Figure 6D). GEPIA2021 platform also revealed the consistent results that NR1H3 is highly expressed in macrophages in BRCA tumor/BRCA normal from TCGA and breast tissue from GTEx using EPIC algorithm (Figure 6E).

We further analyzed the correlation of NR1H3 expression and monocyte/macrophage markers in tumor tissues using TIMER2. We adjusted these results based on tumor purity, and revealed significant correlations between NR1H3 expression and monocyte markers (CD86, CD115/CSF1R, CD14), macrophage markers (CCL2, CD68, IL10, CD80), M2 macrophage markers (CD163, VSIG4, MS4A4A) and M1 macrophage markers (IRF5), whereas the M1 macrophage markers INOS/NOS2 and COX2/PTGS2 showed no correlation with NR1H3 expression (Supplementary Figure S6A).

**FIGURE 7:**
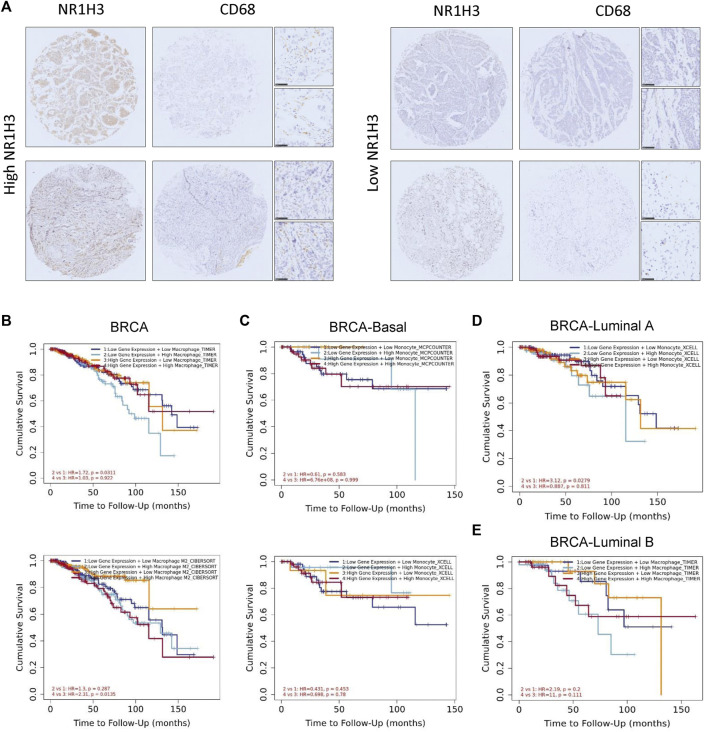
The survival curves, featuring patterns of NR1H3 gene expression and macrophage infiltration level were shown on the diagram. The association between macrophages and OS was displayed as the low or high expression of NR1H3. **(A)** Representative IHC analysis of NR1H3 and CD68, Scale bar = 50 μm. **(B)** Kaplan-Meier curve for the NR1H3 expression level and macrophage infiltration using TIMER algorithms in BRCA. **(C)** Kaplan-Meier curve for the NR1H3 expression level and monocyte infiltration using MCPCOUNTER algorithm in basal breast cancer. **(D)** Kaplan-Meier curve for the NR1H3 expression level and monocyte infiltration using XCELL algorithm in luminal A breast cancer. **(E)** Kaplan-Meier curve for the NR1H3 expression level and macrophage infiltration using TIMER algorithm in luminal B breast cancer. OS, overall survival; IHC, immunohistochemical.

In order to verify the findings from the database, we detected the protein level of NR1H3 and macrophage marker CD68 in paraffin tissue microarrays from breast cancer patients by IHC. The slides showed that NR1H3 and CD68 protein were expressed in interstitial cells of breast tumor tissues. A typical staining pattern is shown in Figure 7A. However, we did not find a linear relationship between NR1H3 and CD68 expression levels.

### NR1H3 expression and macrophage infiltration are independent risk factors for BRCA

As mentioned above, we observed a statistical positive correlation between the immune infiltration of macrophages and NR1H3 expression in BRCA. Then we evaluated the prognostic efficiency of the combination of infiltrated macrophages and NR1H3 expression patterns for breast cancer (Supplementary Figure S6B). The low expression of NR1H3 accompanied by a high level of infiltrated macrophages was associated with poor prognosis in BRCA. However, there was no significant relations between the B Cells/CD4+ T Cells/CD8+ T Cells/neutrophils/NK cells/DCs and prognosis under the low expression level of NR1H3 based on most algorithms (Supplementary Figure S7). Specifically, under low NR1H3 expression, higher macrophage infiltration level had a worse outcome in BRCA using the TIMER algorithm (HR = 1.72, *p* = 0.0311), compared with lower macrophage infiltration level. On the contrary, the low M2 macrophage infiltration level predicted favorable prognosis under the high expression of NR1H3 using the CIBERSORT algorithm in BRCA (HR = 2.31, *p* = 0.0135), compared with the high M2 macrophage infiltration level. In BRCA-LumA, under low NR1H3 expression, higher monocyte level had a worse outcome (HR = 3.12, *p* = 0.0279). The statistically different scatterplot data of the above tumors produced using different algorithms was presented in Figures 7B–E; Supplementary Figure S8.

Additionally, to evaluate whether NR1H3 expression level and macrophage infiltration are independent risk factors for prognosis of BRCA, we conducted the univariate and multivariate analysis included seven variables: macrophage infiltration level, age, stage, gender, race, tumor purity and expression of NR1H3 (Table 2). The results showed that macrophage infiltration (HR = 6.20, *p* = 0.002), stage 3 (HR = 3.11, *p* = 0), stage 4 (HR = 13.17, *p* = 0) and NR1H3 expression (HR = 0.75, *p* = 0.018) were prognostic variables for the prognosis of OS in BRCA patients. After adjustments of age, stage, gender, race, and tumor purity, the level of macrophage infiltration (HR = 8.44, *p* = 0.002) and the expression of NR1H3 (HR = 0.73, *p* = 0.044) were independent prognostic factors in BRCA. These results suggest that NR1H3 is an independent prognostic biomarker and combining its expression level with the macrophage would help to play a more effective role in the prognosis prediction of BRCA.

**TABLE 2:** Univariate and multivariate analysis of prognostic variables of OS in BRCA.

Variables	Univariate analysis			Multivariate analysis		
	HR	95%CI	*p*-value	HR	95%CI	*p*-value
Macrophage	6.2	1.93–19.91	**0.002**	8.44	2.1–32.66	**0.002**
Age	1.03	1.02–1.04	**0**	1.04	1.02–1.05	**0**
Stage2	1.66	0.96–2.86	0.068	1.47	0.81–2.67	0.204
Stage3	3.11	1.75–5.51	**0**	3.27	1.77–6.03	**0**
Stage4	13.17	6.49–26.73	**0**	13.48	6.29–28.92	**0**
Gender male	0.83	0.12–5.97	0.857	0.97	0.14–7.02	0.978
Race Black	1.61	0.49–5.28	0.436	1.05	0.31–3.55	0.935
Race White	1.35	0.43–4.26	0.61	0.72	0.22–2.33	0.587
Purity	1.68	0.8–3.52	0.171	1.21	0.5–2.91	0.675
NR1H3	0.75	0.59–0.95	**0.018**	0.73	0.54–0.99	**0.044**

OS, overall survival; BRCA, breast invasive carcinoma.

*p*-values < 0.05 are displayed in bold.

### Association between NR1H3 and immunomodulatory molecules

The TISIDB database was used to infer the correlations between expression of NR1H3 and immunomodulators/chemokines across human cancers. As shown in Supplementary Figure S9, the relations between immunoinhibitors, immunostimulators, MHC molecules, chemokines and chemokine receptors and expression of NR1H3 were strongly correlated. Furthermore, NR1H3 was also positively associated with immune checkpoint molecules (PD-1/CD274, PD-L1/PDCD1, PD-L2/PDCD1LG2, and CTLA-4) in TIMER2 database (Supplementary Figure S10). These results suggest that NR1H3 is closely related to the immune status of human cancers.

### Gene interaction network of NR1H3 and functional enrichment analysis of NR1H3-related partners

**FIGURE 8:**
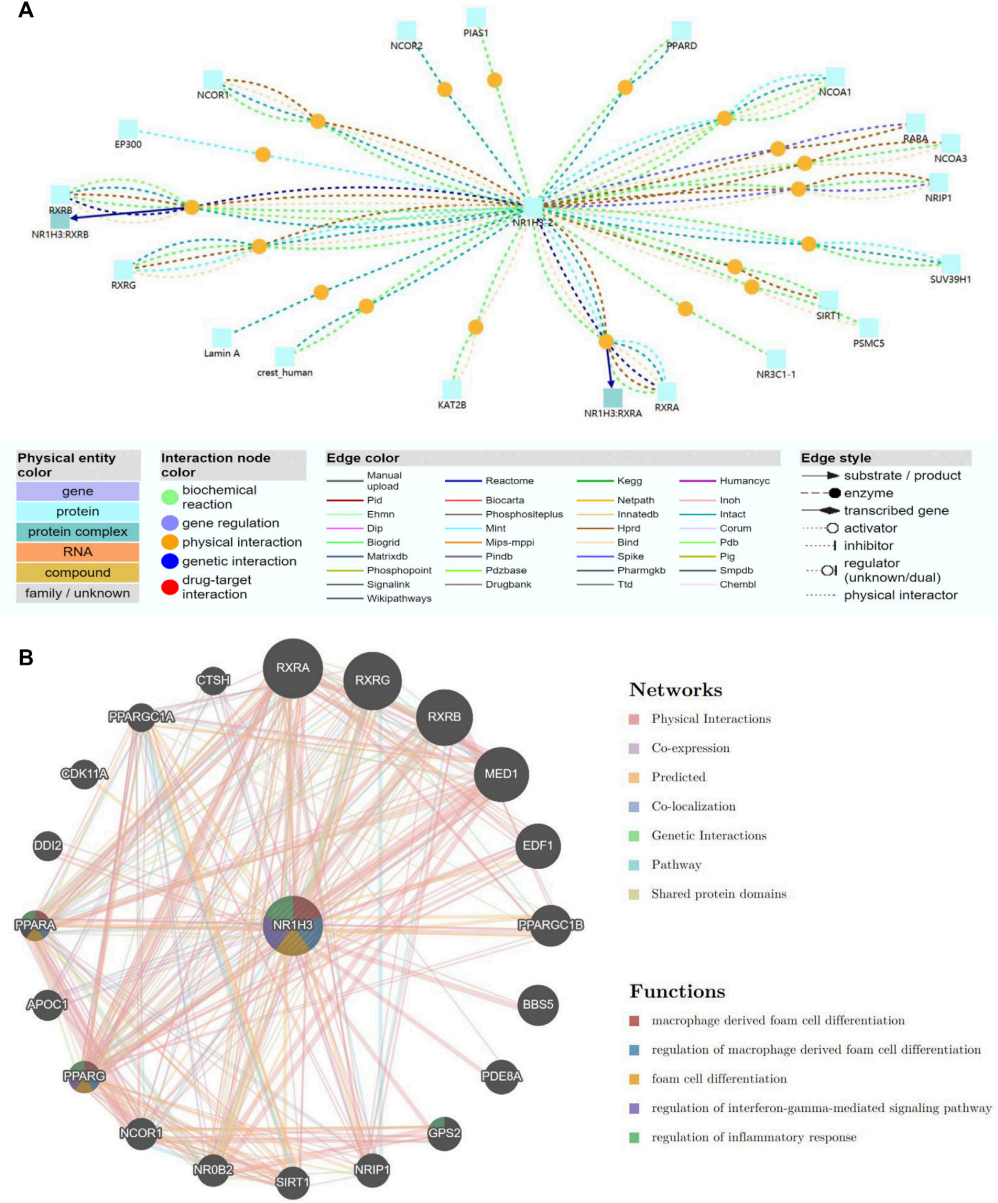
Gene interaction network of NR1H3. **(A)** The gene interaction network of NR1H3 using the ConsensusPathDB database. **(B)** The gene interaction network of NR1H3 constructed by the GeneMANIA.

To understand the biological function of NR1H3, ConsensusPathDB was used to integrate interaction network of NR1H3 in *Homo sapiens*. The network defined the neighborhood-based entity set centered by NR1H3 and containing 19 interaction nodes and 22 physical entity nodes (Figure 8A; Supplementary Figure S11A).

A gene interaction network was constructed using the GeneMANIA. Twenty NR1H3-associated genes were observed in the interaction network, functions of which focused on macrophage derived foam cell differentiation, regulation of macrophage derived foam cell differentiation, foam cell differentiation, regulation of interferon-gamma-mediated signaling pathway and regulation of inflammatory response (Figure 8B).

To further investigate the molecular mechanism of the NR1H3 in tumorigenesis, we attempted to screen out the targeting NR1H3-binding proteins and the NR1H3 expression-correlated genes for a series of pathway enrichment analyses. Based on the STRING tool, we obtained a total of 152 NR1H3-binding proteins, which were supported by experimental evidence. We used the GEPIA2 tool to combine all tumor expression data of TCGA and obtained the top 300 genes that correlated with NR1H3 expression. An intersection analysis of the above two groups showed two common members, ITGB2 and ITGB7 (Supplementary Figure S11B–D).

We also analyzed a gene interaction network of NR1H3 and common member ITGB2 using the GeneMANIA. The functions of phagocytosis, toll-like receptor signaling pathway, regulation of innate immune response, macrophage activation, regulation of toll-like receptor 4 signaling pathway, positive regulation of immune effector process and positive regulation of innate immune response were significantly related (Supplementary Figure S12).

### Relationship between mutation status and NR1H3 expression in breast cancer

**FIGURE 9:**
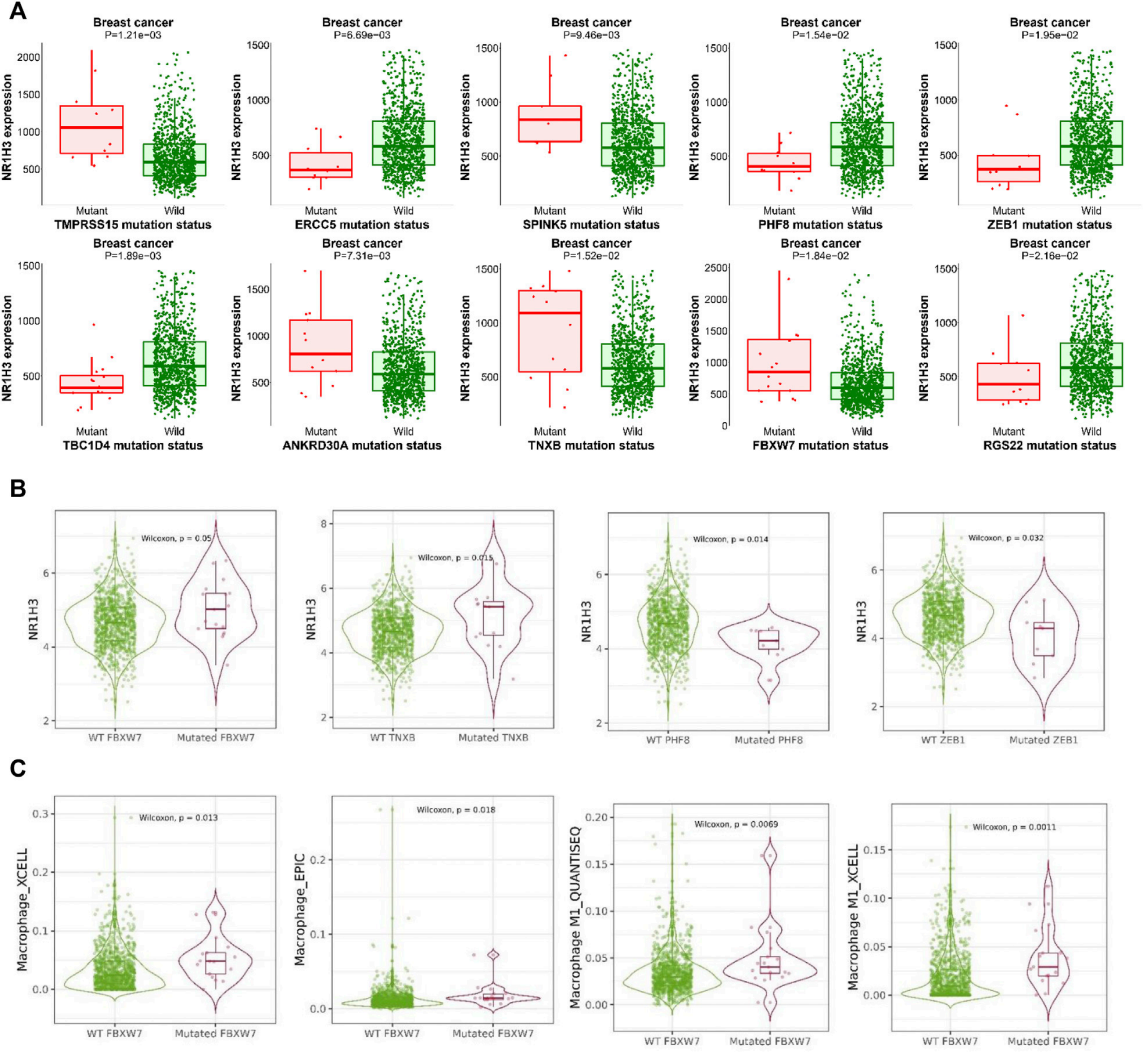
The relationship between gene mutation status and NR1H3 expression in breast cancer. **(A)** The mutant genes that affect the expression of NR1H3 gene in the muTarget database. **(B)** The relationship between gene mutation status and NR1H3 expression in breast cancer was verified using TIMER2. **(C)** Higher infiltration of macrophage was shown in the FBXW7 mutant group, compared with the wild type group.

In order to reveal the relationship between gene mutation status and NR1H3 expression in breast cancer, we screened mutations resulting in NR1H3 expression change using muTarget tool. The results showed that mutations of TMPRSS15, TBC1D4, ERCC5, ANKRD30A, SPINK5, TNXB, PHF8, FBXW7, ZEB, and RGS22 would lead to the alteration of NR1H3 expression (Figure 9A). Then we verified the above genes in the TIMER2 database and found that the FBXW7 mutation was significantly associated with high NR1H3 expression and high macrophage infiltration (Figure 9B, Figure 9C, Supplementary Figure S13A,B). Higher infiltration of M1 macrophage and lower infiltration of M2 macrophage were shown in the mutant group, compared with the wild type group (Figure 9C; Supplementary Figure S13C).

### Association between NR1H3 and response to drug therapy

We used ROC Plotter to identify whether NR1H3 predicted benefit from endocrine therapy and chemotherapy. ROC Plotter showed that NR1H3 was upregulated in responders of luminal A (AUC = 0.564, *p* = 2.9e-02) and grade 1 subtype breast cancer patients with Tamoxifen treatment (AUC = 0.822, *p* = 1.1e-05) based on relapse-free survival (RFS) at 5 years (Supplementary Figure S14A). For pathological response, high NR1H3 expression predicted benefit from Anthracycline treatment in TNBC, luminal A, HER2 negative, ER negative, grade 1 and nodal positive subtype patients (Supplementary Figures S14B,C). NR1H3 was upregulated in responders of HER2 negative, ER negative, grade 1, nodal positive subtype patients with Taxane treatment (Supplementary Figure S15A). For patients treated with FAC (Fluorouracil, Adriamycin, Cytoxan), high NR1H3 expression predicted pathological response in luminal A and HER2 negative breast cancer (Supplementary Figure S15B). In addition, NR1H3 was highly expressed in pathological responders receiving FEC (Fluorouracil, Epirubicin, Cyclophosphamide) treatment in grade 1 breast cancer patients and Ixabepilone treatment in HER2 negative patients (Supplementary Figures S15C,D). These data indicated that NR1H3 may function as a predictor of chemoresponsiveness in breast cancer.

## Discussion

Previous studies have shown that the nuclear receptor (NR) family members are key regulators of macrophage function, controlling transcriptional programs involved in inflammation and lipid homeostasis (Cully, 2018). As an important member of NR1 subfamily, the role of NR1H3 in tumor microenvironment remains to be revealed. Here, we evaluated the association between NR1H3 expression level and breast cancer patients’ prognosis in multiple public databases. The current clinical data-based evidence supports the role of NR1H3 expression in the clinical features of breast cancer. To our knowledge, this is the first study to report a consistent association between decreasing NR1H3 expression level and poor prognosis in breast cancer patients.

Breast cancer is a heterogeneous disease and is divided into different subtypes based on the expression status of ER/PR and HER-2 (Rakha et al., 2010). Basal tumors, with an overlap in definition with triple-negative subtypes, tends to have a higher relapse risk and is more aggressive than other subtypes (Colombo et al., 2011; Valentin et al., 2012). Luminal A tumors had the lowest rate of relapse when comparing other subtypes (Wang et al., 2011). We observed that low expression of NR1H3 was found to be significantly associated with poor clinical outcome in basal subtype, HER2 positive subtype and grade 3 breast cancer patients. Moreover, NR1H3 expression and macrophage infiltration level were indicated as novel prognostic indicators for breast cancer, conferring significantly worse survival for those with low NR1H3 expression accompanied by a high level of infiltrated macrophages.

Our data are in line with experimental results previously published.NR1H3 was reported to be an onco-suppressor gene in various cancers (Vigushin et al., 2004; Garattini et al., 2016; Wu et al., 2017; Cully, 2018). *In vitro*, culture medium from NR1H3 activated macrophages causes growth inhibition and apoptosis of breast tumor cells (El Roz et al., 2013). In mouse models, NR1H3 ligands augments mammary-tumor growth and increases NR1H3-dependent metastasis (Nelson et al., 2014). These findings indicate that NR1H3 may be important in breast carcinogenesis. Whether pharmacological NR1H3 agonists have potential preventive or therapeutic antitumor activity in breast cancer needs more studies to confirm.

To further explore the underlying mechanisms of NR1H3 in breast carcinogenesis, we investigated the correlation of NR1H3 expression with tumor-infiltrating immune cells of breast cancer. Our results revealed the important role of NR1H3 in TME as well as providing a potential relationship between NR1H3 and tumor-immune interactions in breast cancer. As we all known, activated immune cells attacks tumor cells to prevent the development of cancer in the early phase of carcinogenesis. Here, we provide evidence that high expression of NR1H3 is strongly correlated with multiple immune infiltration in breast cancer tissues, including B Cells, CD4^+^ T Cells, CD8^+^ T Cells, neutrophils, macrophages and DCs. These results indicate that expression of NR1H3 is related to the immune activation of TME.

Existing studies have shown that tumor-infiltrating immune cells play important roles in the initiation, progression, metastasis and therapeutic resistance of cancers (Fridman et al., 2011; Gajewski et al., 2013; Quail and Joyce, 2013; Topalian et al., 2015; Steven and Seliger, 2018). Among various infiltrating immune cells, high macrophages infiltrate density predicts worse patient prognosis. Intratumoral macrophage populations can be classified as M1 and M2 macrophages along a functional scale. The M1 macrophages exhibit antitumor activity by releasing pro-inflammatory cytokines, oxygen intermediates and reactive nitrogen. In contrast, the M2 macrophages are stimulated by the Th2 cytokines to exert protumor ability, and can participate in carcinogenesis in several ways, including metastasis, immune escape and angiogenesis (Li X. et al., 2017; De Palma et al., 2017; Cully, 2018; Zhu et al., 2019). In the present study, we showed that NR1H3 was correlated with infiltrating level of macrophages as well as the expression of monocyte/macrophage markers in breast cancer. NR1H3 mainly expressed in monocytes/macrophages and high amplification of NR1H3 was associated with a low M2 macrophage infiltration level. Based on these results, we evaluated the prognostic efficiency of the combination of infiltrated macrophages and NR1H3 expression patterns for breast cancer. As we expected, the low expression of NR1H3 and low M1 (anti-tumor)/high M2 (pro-tumor) macrophage infiltration predicted a poor prognosis in breast cancer patients.

We found the association between NR1H3 expression and mutated FBXW7, which is one of the most frequently mutated genes in human cancers and its functional inactivation can lead to tumorigenesis. FBXW7α, the most abundant isoform in proliferating cells, attenuates the LPS response through inhibition of C/EBPδ and TLR4 expression and that FBXW7α-depletion alone is sufficient to activate inflammatory signaling (Balamurugan et al., 2013). Importantly, FBXW7α plays a negative role in TAM M1 polarization, and FBXW7α siRNA increases the expression of M1 markers, including the secretion of TNF-α, IL-12, and IL-6, and COX2 and NOS2 expression in the cytoplasm. Long et al. proved that the FBXW7α/miR-205 axis might regulate TAM polarization by affecting SMAD1 expression. (Long and Zhu, 2019). In our results, FBXW7 mutation is related to up-regulation of NR1H3 expression, high M1 macrophage infiltration and low M2 macrophage infiltration, which is consistent with our previous results of NR1H3.

We also integrated the information on NR1H3-binding components and NR1H3 expression-related genes for a series of enrichment analyses. We identified a potential impact of NR1H3 in regulation of macrophage activation and inflammatory response regulation. Gene interaction network and functional enrichment analysis revealed the molecular mechanism by which low expression of NR1H3 gene leads to poor prognosis of breast cancer patients. However, the limitations in current study are also lies in the lack of experimental verification. Moreover, the detailed mechanisms of NR1H3 in regulating activation of TME in breast cancer needs further study.

In summary, our study showed that low NR1H3 expression was correlated with worse survival, especially for basal subtype, HER2 positive subtype and grade 3 breast cancer patients. NR1H3 was related to immune cells infiltration and regulation of macrophage activation. Importantly, the expression of NR1H3 and macrophage infiltration level were independent risk factors for prognosis of breast cancer patients. Therefore, NR1H3 could be a useful biomarker in breast cancer patients and activation of NR1H3 might be a potential therapeutic antitumor strategy of breast cancer.

## Data Availability

The original contributions presented in the study are included in the article/supplementary materials, further inquiries can be directed to the corresponding authors.

## Supplementary material

Supplementary Figure S1 Box plots showing lower NR1H3 expression in breast tissues compared with normal tissues. Using the Oncomine, the expression difference of NR1H3 between tumor and adjacent normal tissues was compared in Sorlie Breast (A), Sorlie Breast 2 (B), and Ma Breast 4 (C) datasets. (D) The level of NR1H3 expression in BRCA based on tumor subclasses using the UALCAN database. (E) The level of NR1H3 mRNA expression in different tumor stages using the UALCAN database. BRCA, breast invasive carcinoma.

Supplementary Figure S2 Kaplan-Meier survival curves comparing the high and low expression of NR1H3 in breast cancer. (A, B) Kaplan–Meier survival curves of OS, DFS, and PFS comparing the high and low expression of NR1H3 in the GEPIA2 and GSCA database. (C-I) Kaplan-Meier survival curves of DMFS and PPS comparing the high and low expression of NR1H3 in different subtypes in the TCGA dataset. OS, overall survival; DFS, disease-free survival; PFS, progress free survival; DMFS, distant metastasis-free survival; PPS, post progression survival.

Supplementary Figure S3 The landscape of relation between NR1H3 expression and TILs in different cancer types available at TISIDB database. TILs, tumor-infiltrating lymphocytes.

Supplementary Figure S4 The landscape of relations between NR1H3 expression and immune infiltration of B cells, CD4+ T cells, CD8+ T cells, monocytes, macrophages, DCs, neutrophils and NK cells across human cancers in the TIMER database. DCs, dendritic cells; NK, natural killer.

Supplementary Figure S5 Correlation between NR1H3 expression and the TME. (A, B) Correlation analysis between the expression of NR1H3 in primary breast cancer tissues and the TME. TME, tumor microenvironment.

Supplementary Figure S6 Correlation and prognostic efficiency of macrophage infiltration across multiple cancer types. (A) Correlation between NR1H3 expression and gene markers of monocyte/macrophage across multiple cancer types. (B) The clinical relevance of the combination of infiltrated macrophages and NR1H3 expression patterns across multiple cancer types.

Supplementary Figure S7 Assessment of association between the combination of immune infiltrates and NR1H3 expression patterns and clinical outcome across diverse cancer types.

Supplementary Figure S8 Kaplan-Meier curves for NR1H3 expression level and monocyte/macrophage infiltration using multiple algorithms in BRCA (A), basal subtype (B), luminal A subtype (C) and luminal B subtype (D). BRCA, breast invasive carcinoma.

Supplementary Figure S9 The landscape of relationship between NR1H3 expression and immunoinhibitors (A), immunostimulators (B), MHC molecules (C), chemokines (D) and chemokine receptors (E) across human cancers.

Supplementary Figure S10 Correlation of NR1H3 expression and PD-1/CD274, PD-L1/PDCD1, PD-L2/PDCD1LG2, and CTLA-4 across diverse human cancers.

Supplementary Figure S11 Genes correlated with NR1H3. (A) Detailed information of NR1H3 interaction networks. The network consisted of 19 physical interactions from eight different databases. (B) An intersection analysis of the NR1H3-binding and correlated genes was conducted. (C) Correlation of NR1H3 expression and ITGB2 in BRCA using TIMER2 database. (D) Correlation of NR1H3 expression and ITGB7 in BRCA using TIMER2 database. BRCA, breast invasive carcinoma.

Supplementary Figure S12 The gene interaction network of NR1H3 and ITGB2 constructed by the GeneMANIA.

Supplementary Figure S13 The relationship between gene mutation status and NR1H3 expression in breast cancer. (A) The relationship between FBXW7 and NR1H3 expression in breast cancer was verified using GEPIA database. (B) The relationship between TMPRSS15 and RGS22 gene mutation status and NR1H3 expression in breast cancer was verified using TIMER2. (C) The FBXW7 mutation was significantly associated with lower infiltration of M2 macrophage.

Supplementary Figure S14 Association between NR1H3 and response to drug therapy. (A) Association between NR1H3 and response to Tamoxifen treatment based on RFS at 5 years. (B, C) Association between NR1H3 and pathological response for Anthracycline treatment. RFS, relapse-free survival.

Supplementary Figure S15 Association between NR1H3 and pathological response for Taxane (A), FAC (B), FEC (C) and Ixabepilone (D) treatment in breast cancer patients. FAC, Fluorouracil, Adriamycin, Cytoxan; FEC, Fluorouracil, Epirubicin, Cyclophosphamide.

## References

[B1] Balamurugan K. Sharan S. Klarmann K. D. Zhang Y. Coppola V. Summers G. H. (2013). FBXW7α attenuates inflammatory signalling by downregulating C/EBPδ and its target gene Tlr4. Nat. Commun. 4, 1662. 10.1038/ncomms2677 23575666 PMC3625980

[B2] Bensinger S. J. Bradley M. N. Joseph S. B. Zelcer N. Janssen E. M. Hausner M. A. (2008). LXR signaling couples sterol metabolism to proliferation in the acquired immune response. Cell 134, 97–111. 10.1016/j.cell.2008.04.052 18614014 PMC2626438

[B3] Biglia N. D’Alonzo M. Sgro L. G. Tomasi Cont N. Bounous V. Robba E. (2016). Breast cancer treatment in mutation carriers: surgical treatment. Minerva Ginecol. 68, 548–556. 26822896

[B4] Bray F. Ferlay J. Soerjomataram I. Siegel R. L. Torre L. A. Jemal A. (2018). Global cancer statistics 2018: GLOBOCAN estimates of incidence and mortality worldwide for 36 cancers in 185 countries. CA Cancer J. Clin. 68, 394–424. 10.3322/caac.21492 30207593

[B5] Chandrashekar D. S. Bashel B. Balasubramanya S. A. H. Creighton C. J. Ponce-Rodriguez I. Chakravarthi B. V. S. K. (2017). UALCAN: a portal for facilitating tumor subgroup gene expression and survival analyses. Neoplasia 19, 649–658. 10.1016/j.neo.2017.05.002 28732212 PMC5516091

[B6] Coleman M. Quaresma M. Berrino F. Lutz J. M. Angelis R. Capocaccia R. (2008). Cancer survival in five continents: a worldwide population-based study (CONCORD). Lancet Oncol. 9, 730–756. 10.1016/S1470-2045(08)70179-7 18639491

[B7] Colombo P. E. Milanezi F. Weigelt B. Reis-Filho J. S. (2011). Microarrays in the 2010s: The contribution of microarray-based gene expression profiling to breast cancer classification, prognostication and prediction. Breast Cancer Res. 13, 212. 10.1186/bcr2890 21787441 PMC3218943

[B8] Cully M. (2018). Cancer: re-educating tumour-associated macrophages with nanoparticles. Nat. Rev. Drug Disc 17, 468. 10.1038/nrd.2018.102 29904193

[B9] De Palma M. Biziato D. Petrova T. V. (2017). Microenvironmental regulation of tumour angiogenesis. Nat. Rev. Cancer 17, 457–474. 10.1038/nrc.2017.51 28706266

[B10] Duc D. Vigne S. Pot C. (2019). Oxysterols in autoimmunity. Int. J. Mol. Sci. 20, 4522. 10.3390/ijms20184522 31547302 PMC6770630

[B11] El Roz A. Bard J. M. Valin S. Huvelin J. M. Nazih H. (2013). Macrophage apolipoprotein E and proliferation of MCF-7 breast cancer cells: role of LXR. Anticancer Res. 33, 3783–3789. 24023310

[B12] Fekete J. T. Győrffy B. (2019). ROCplot.org: Validating predictive biomarkers of chemotherapy/hormonal therapy/anti-HER2 therapy using transcriptomic data of 3, 104 breast cancer patients. Int. J. Cancer 145, 3140–3151. 10.1002/ijc.32369 31020993

[B13] Fessler M. B. (2008). Liver X receptor: Crosstalk node for the signaling of lipid metabolism, carbohydrate metabolism, and innate immunity. Curr. Signal Transduct. Ther. 3, 75–81. 10.2174/157436208784223170 24563635 PMC3931522

[B14] Franz M. Rodriguez H. Lopes C. Zuberi K. Montojo J. Bader G. D. (2018). GeneMANIA update 2018. Nucleic Acids Res. 46, W60–W64. 10.1093/nar/gky311 29912392 PMC6030815

[B15] Fridman W. H. Galon J. Dieu-Nosjean M. C. Cremer I. Fisson S. Damotte D. (2011). Immune infiltration in human cancer: Prognostic significance and disease control. Curr. Top. Microbiol. Immunol. 344, 1–24. 10.1007/82_2010_46 20512556

[B16] Gajewski T. F. Schreiber H. Fu Y. X. (2013). Innate and adaptive immune cells in the tumor microenvironment. Nat. Immunol. 14, 1014–1022. 10.1038/ni.2703 24048123 PMC4118725

[B17] Garattini E. Bolis M. Gianni M. Paroni G. Fratelli M. Terao M. (2016). Lipid-sensors, enigmatic-orphan and orphan nuclear receptors as therapeutic targets in breast-cancer. Oncotarget 7, 42661–42682. 10.18632/oncotarget.7410 26894976 PMC5173165

[B18] Gradishar W. J. Anderson B. O. Balassanian R. Blair S. L. Burstein H. J. Cyr A. (2015). Breast cancer version 2.2015. J. Natl. Compr. Cancer Netw. 13, 448–475. 10.6004/jnccn.2015.0060 25870381

[B19] Joseph S. B. Bradley M. N. Castrillo A. Bruhn K. W. Mak P. A. Pei L. (2004). LXR-dependent gene expression is important for macrophage survival and the innate immune response. Cell 119, 299–309. 10.1016/j.cell.2004.09.032 15479645

[B20] Kamburov A. Wierling C. Lehrach H. Herwig R. (2009). ConsensusPathDB—a database for integrating human functional interaction networks. Nucleic Acids Res. 37, D623–D628. 10.1093/nar/gkn698 18940869 PMC2686562

[B21] Lanczky A. Nagy A. Bottai G. Munkacsy G. Szabo A. Santarpia L. (2016). miRpower: a web-tool to validate survival-associated miRNAs utilizing expression data from 2178 breast cancer patients. Breast Cancer Res. Treat. 160, 439–446. 10.1007/s10549-016-4013-7 27744485

[B22] Li T. Fu J. Zeng Z. Cohen D. Li J. Chen Q. (2020). TIMER2.0 for analysis of tumor-infiltrating immune cells. Nucleic Acids Res. 48, W509–W514. 10.1093/nar/gkaa407 32442275 PMC7319575

[B23] Li T. Fan J. Wang B. Traugh N. Chen Q. Liu J. S. (2017). TIMER: a web server for comprehensive analysis of tumor-infiltrating immune cells. Cancer Res. 77, e108–e110. 10.1158/0008-5472.CAN-17-0307 29092952 PMC6042652

[B24] Li X. Yao W. Yuan Y. Chen P. Li B. Li J. (2017). Targeting of tumour-infiltrating macrophages via CCL2/CCR2 signalling as a therapeutic strategy against hepatocellular carcinoma. Gut 66, 157–167. 10.1136/gutjnl-2015-310514 26452628

[B25] Long Y. Zhu Y. (2019). Identification of FBXW7α-regulated genes in M1-polarized macrophages in colorectal cancer by RNA sequencing. Saudi Med. J. 40, 766–773. 10.15537/smj.2019.8.24361 31423512 PMC6718864

[B26] Mitro N. Mak P. A. Vargas L. Godio C. Hampton E. Molteni V. (2007). The nuclear receptor LXR is a glucose sensor. Nature 445, 219–223. 10.1038/nature05449 17187055

[B27] Nelson E. R. Chang C. Y. McDonnell D. P. (2014). Cholesterol and breast cancer pathophysiology. Trends Endocrinol. Metab. 25, 649–655. 10.1016/j.tem.2014.10.001 25458418 PMC4268141

[B28] Payne S. J. Bowen R. L. Jones J. L. Wells C. A. (2008). Predictive markers in breast cancer—the present. Histopathology 52, 82–90. 10.1111/j.1365-2559.2007.02897.x 18171419

[B29] Pollard J. W. (2008). Macrophages define the invasive microenvironment in breast cancer. J. Leukoc. Biol. 84, 623–630. 10.1189/jlb.1107762 18467655 PMC2516896

[B30] Quail D. F. Joyce J. A. (2013). Microenvironmental regulation of tumor progression and metastasis. Nat. Med. 19, 1423–1437. 10.1038/nm.3394 24202395 PMC3954707

[B31] Rakha E. A. Reis-Filho J. S. Ellis I. O. (2010). Combinatorial biomarker expression in breast cancer. Breast Cancer Res. Treat. 120, 293–308. 10.1007/s10549-010-0746-x 20107892

[B32] Rhodes D. R. Kalyana-Sundaram S. Mahavisno V. Varambally R. Yu J. Briggs B. B. (2007). Oncomine 3.0: Genes, pathways, and networks in a collection of 18, 000 cancer gene expression profiles. Neoplasia 9, 166–180. 10.1593/neo.07112 17356713 PMC1813932

[B33] Ru B. Wong C. N. Tong Y. Zhong J. Y. Zhong S. S. W. Wu W. C. (2019). TISIDB: an integrated repository portal for tumor-immune system interactions. Bioinformatics 35, 4200–4202. 10.1093/bioinformatics/btz210 30903160

[B34] Steven A. Seliger B. (2018). The role of immune escape and immune cell infiltration in breast cancer. Breast Care (Basel) 13, 16–21. 10.1159/000486585 29950962 PMC6016054

[B35] Sun D. Wang J. Han Y. Dong X. Ge J. Zheng R. (2021). TISCH: a comprehensive web resource enabling interactive single-cell transcriptome visualization of tumor microenvironment. Nucleic Acids Res. 49, D1420–D1430. 10.1093/nar/gkaa1020 33179754 PMC7778907

[B36] Tang Z. Kang B. Li C. Chen T. Zhang Z. (2019). GEPIA2: an enhanced web server for large-scale expression profiling and interactive analysis. Nucleic Acids Res. 47, W556–W560. 10.1093/nar/gkz430 31114875 PMC6602440

[B37] Topalian S. L. Drake C. G. Pardoll D. M. (2015). Immune checkpoint blockade: a common denominator approach to cancer therapy. Cancer Cell 27, 450–461. 10.1016/j.ccell.2015.03.001 25858804 PMC4400238

[B38] Valentin M. D. da Silva S. D. Privat M. Alaoui-Jamali M. Bignon Y. J. (2012). Molecular insights on basal-like breast cancer. Breast Cancer Res. Treat. 134, 21–30. 10.1007/s10549-011-1934-z 22234518

[B39] Vedin L. L. Lewandowski S. A. Parini P. Gustafsson J. A. Steffensen K. R. (2009). The oxysterol receptor LXR inhibits proliferation of human breast cancer cells. Carcinogenesis 30, 575–579. 10.1093/carcin/bgp029 19168586

[B40] Vigushin D. M. Dong Y. Inman L. Peyvandi N. Alao J. P. Sun C. (2004). The nuclear oxysterol receptor LXRalpha is expressed in the normal human breast and in breast cancer. Med. Oncol. 21, 123–131. 10.1385/MO:21:2:123 15299184

[B41] Wang Y. Yin Q. Yu Q. Zhang J. Liu Z. Wang S. (2011). A retrospective study of breast cancer subtypes: The risk of relapse and the relations with treatments. Breast Cancer Res. Treat. 130, 489–498. 10.1007/s10549-011-1709-6 21837481

[B42] Wu J. Wan F. Sheng H. Shi G. Shen Y. Lin G. (2017). NR1H3 expression is a prognostic factor of overall survival for patients with muscle‐invasive bladder cancer. J. Cancer 8, 852–860. 10.7150/jca.17845 28382148 PMC5381174

[B43] Yu X. Guo J. Zhou Q. Huang W. Xu C. Long X. (2021). A novel immune-related prognostic index for predicting breast cancer overall survival. Breast Cancer 28, 434–447. 10.1007/s12282-020-01175-z 33146847

[B44] Zelcer N. Hong C. Boyadjian R. Tontonoz P. (2009). LXR regulates cholesterol uptake through Idol-dependent ubiquitination of the LDL receptor. Sci. (New York, NY) 325, 100–104. 10.1126/science.1168974 PMC2777523 19520913

[B45] Zhu Y. Yang J. Xu D. Gao X. M. Zhang Z. Hsu J. L. (2019). Disruption of tumour-associated macrophage trafficking by the osteopontin-induced colony-stimulating factor-1 signalling sensitises hepatocellular carcinoma to anti-PD-L1 blockade. Gut 68, 1653–1666. 10.1136/gutjnl-2019-318419 30902885

