# Supplementary figures and images for: Low expression of NR1H3 correlates with macrophage infiltration and indicates worse survival in breast cancer
Source: Front Genet. 2023 Jan 9;13:1067826. doi: 10.3389/fgene.2022.1067826 (PMC9868774; doi:10.3389/fgene.2022.1067826)

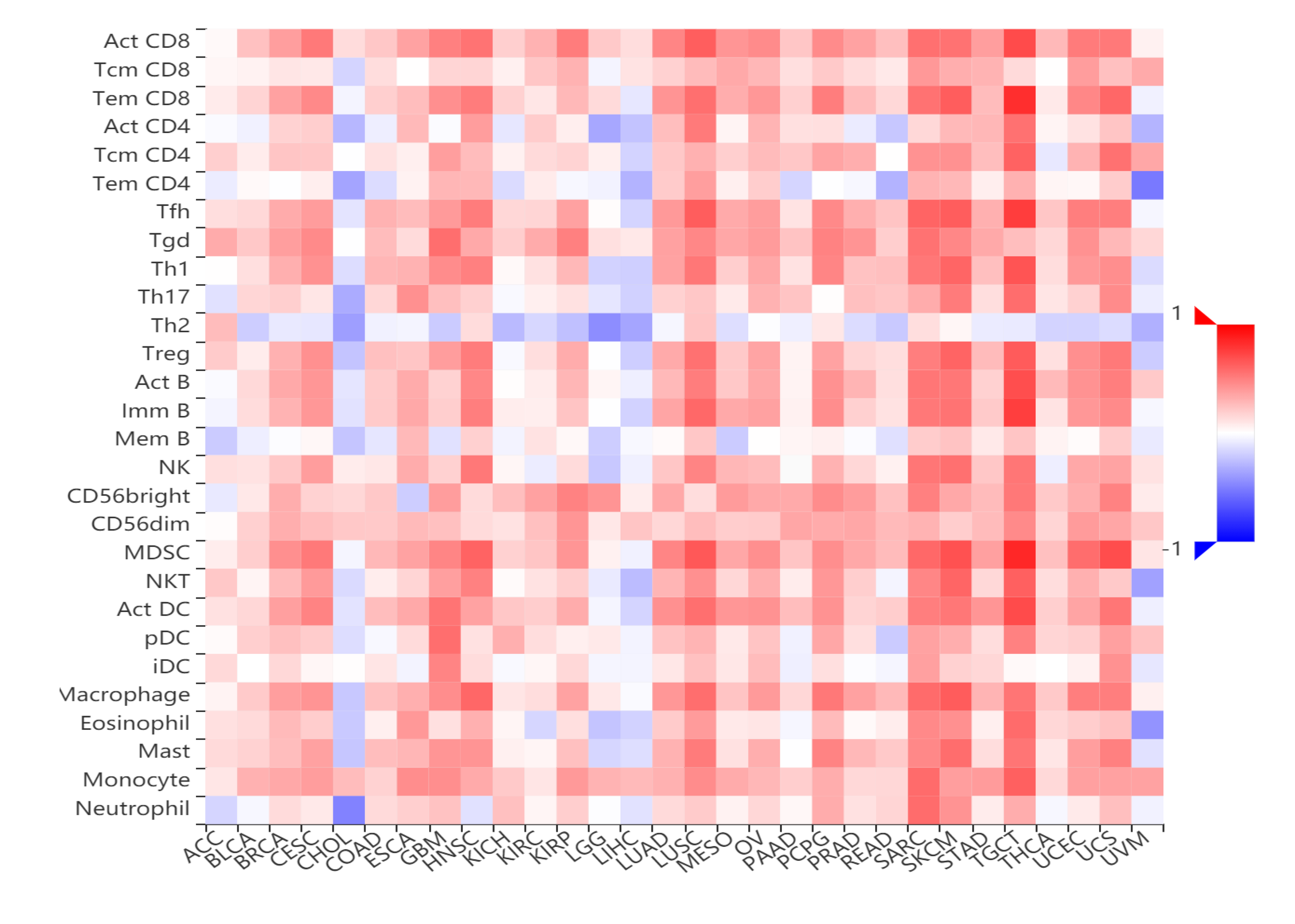

Supplement: Supplementary file 1 [file Image3.TIFF]

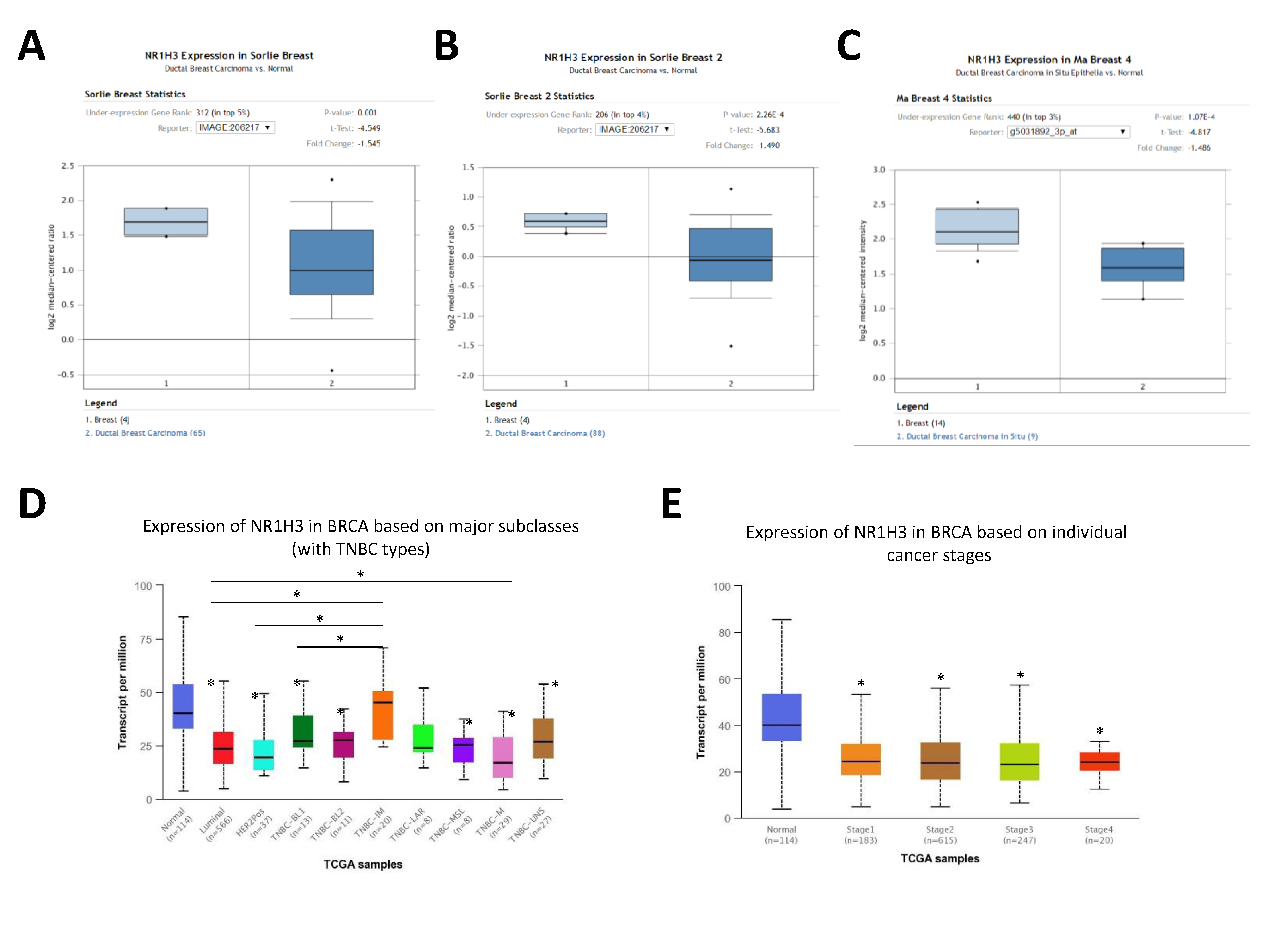

Supplement: Supplementary file 2 [file Image1.TIFF]

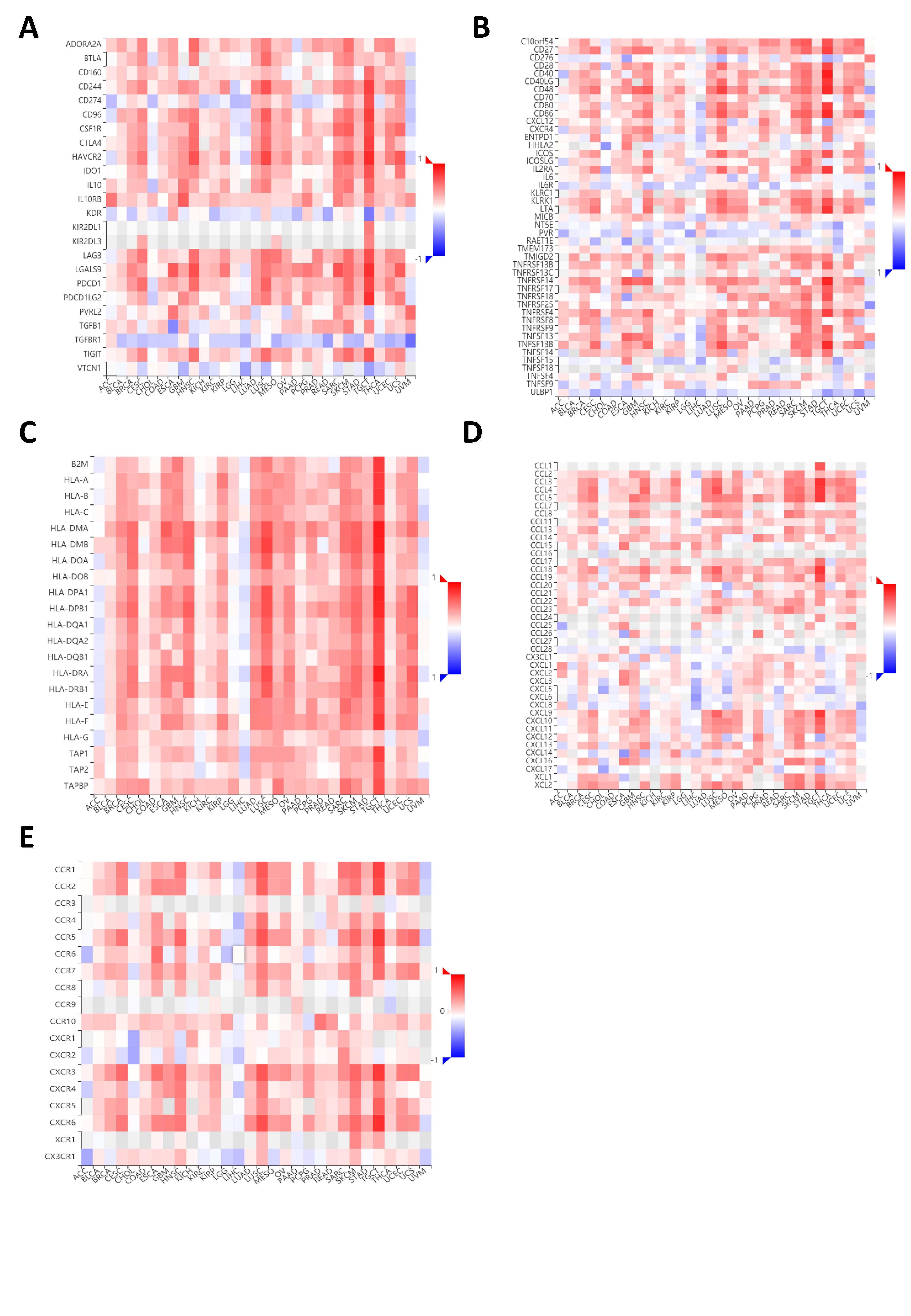

Supplement: Supplementary file 3 [file Image9.TIFF]

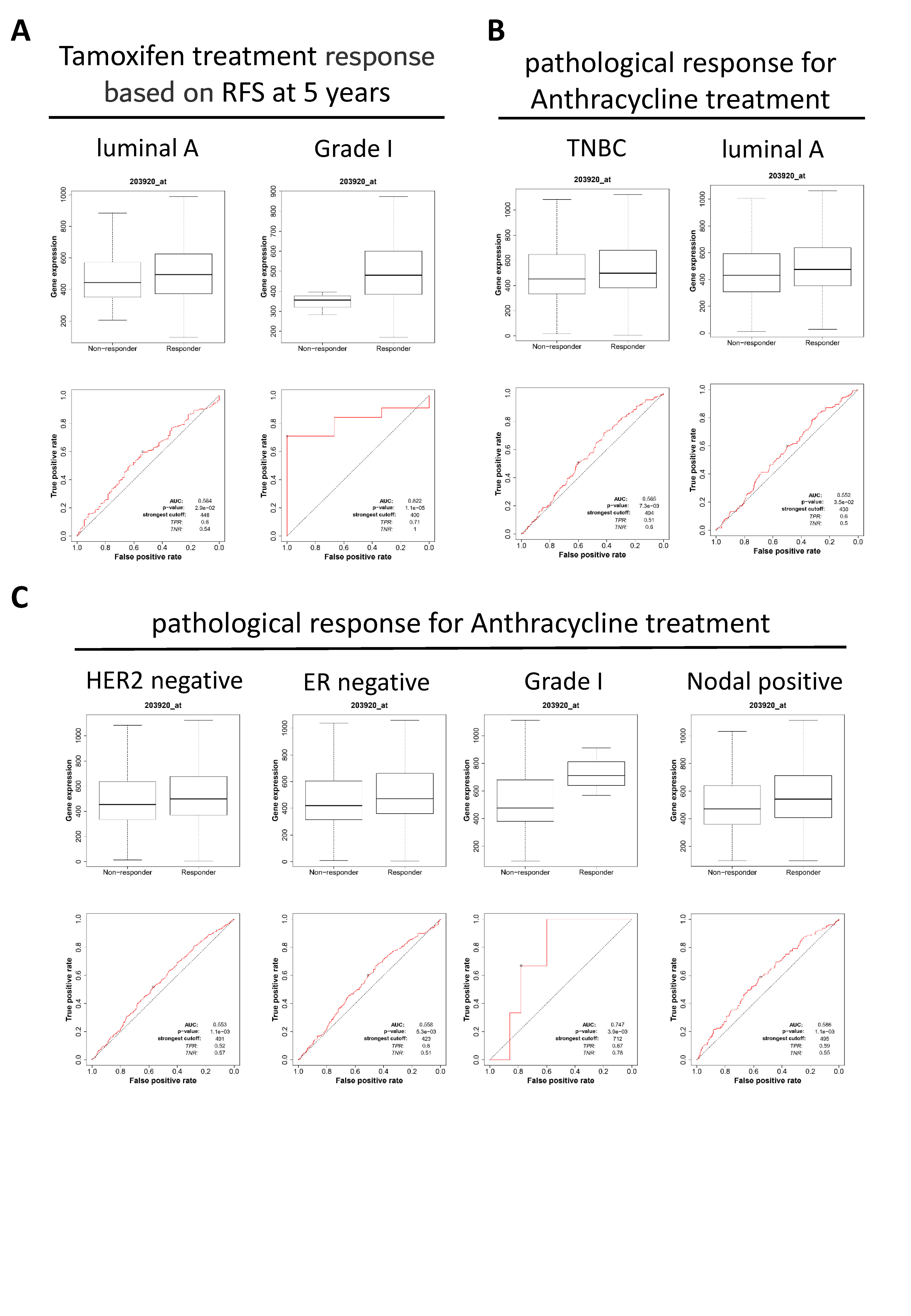

Supplement: Supplementary file 4 [file Image14.TIFF]

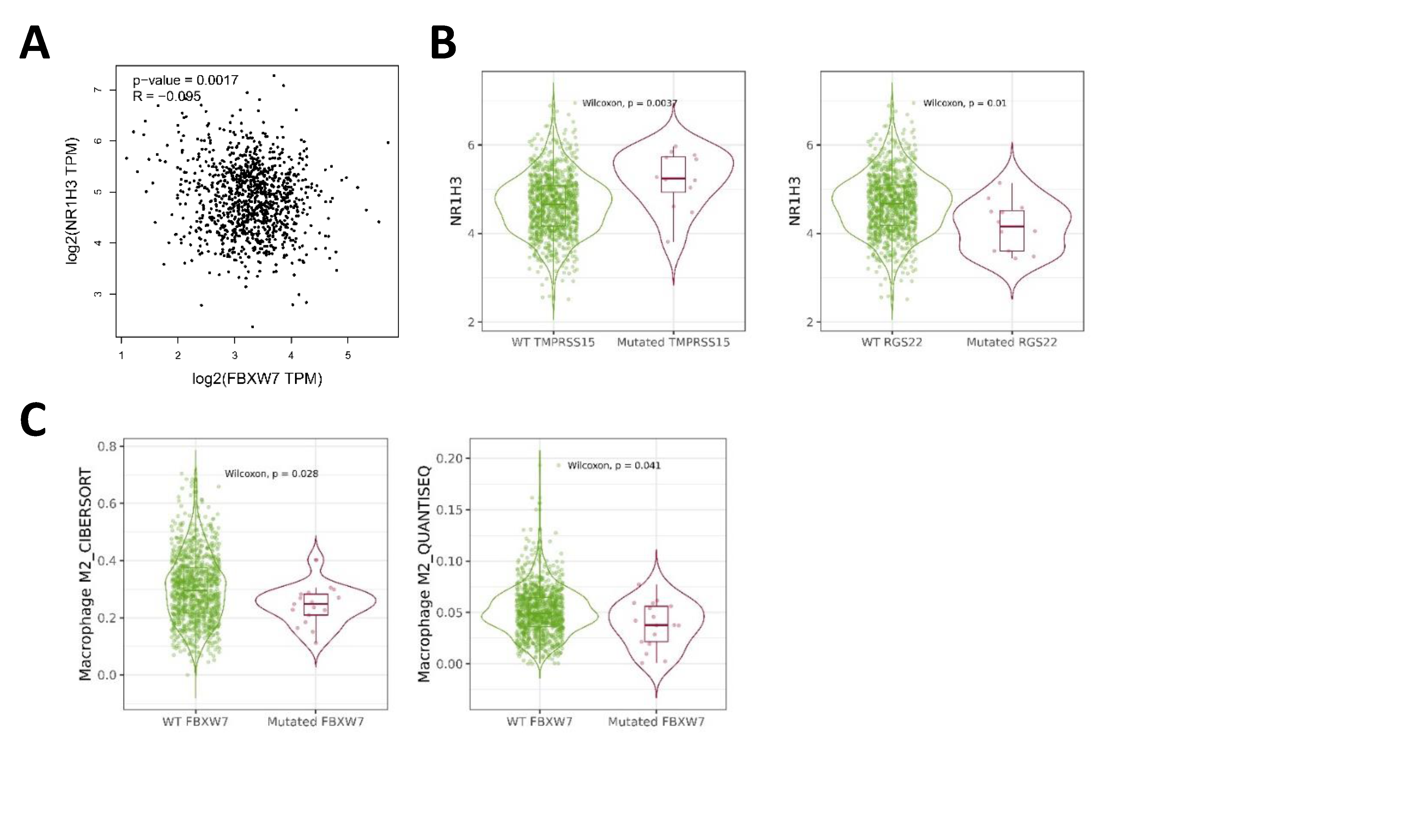

Supplement: Supplementary file 5 [file Image13.TIFF]

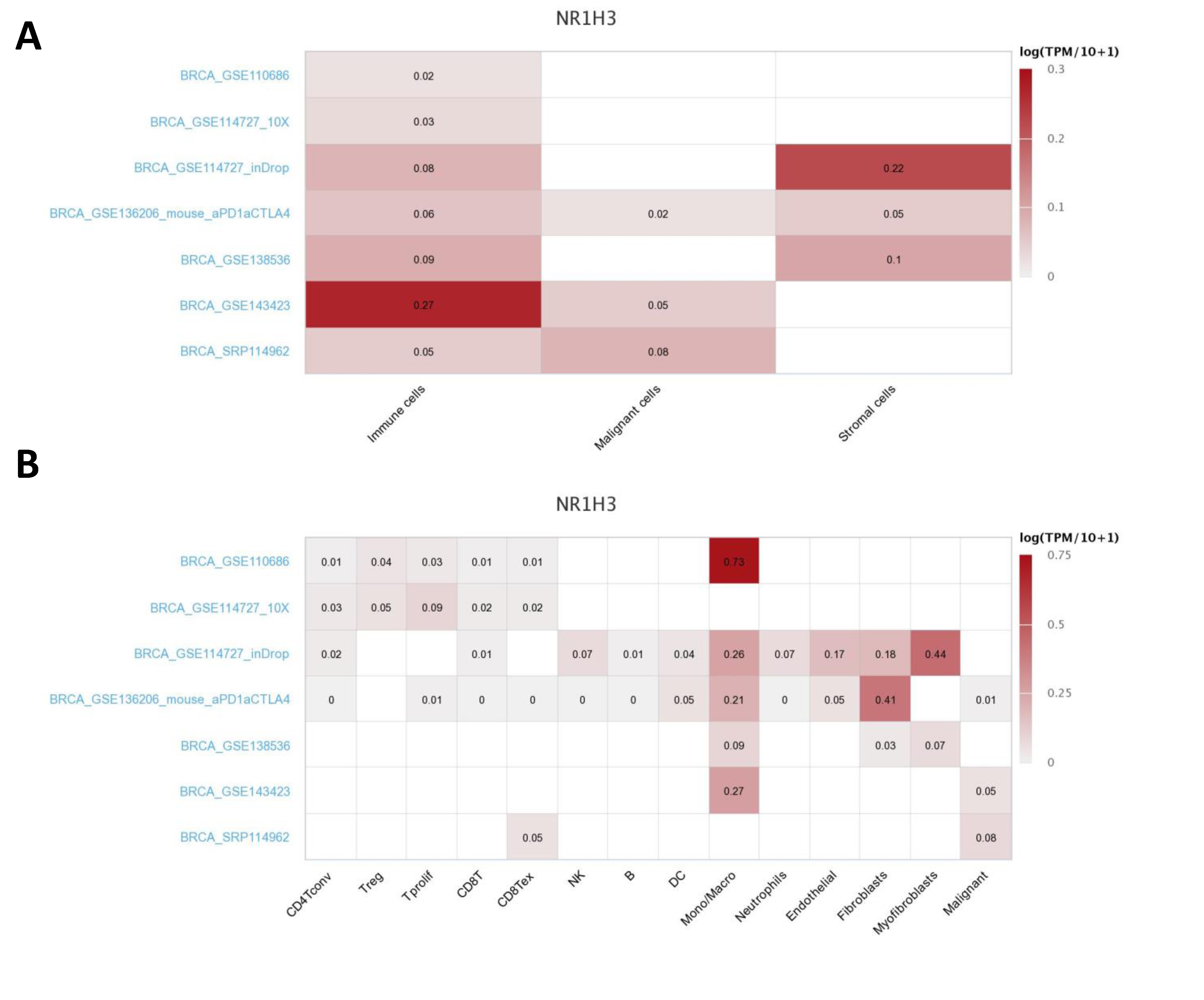

Supplement: Supplementary file 6 [file Image5.TIFF]

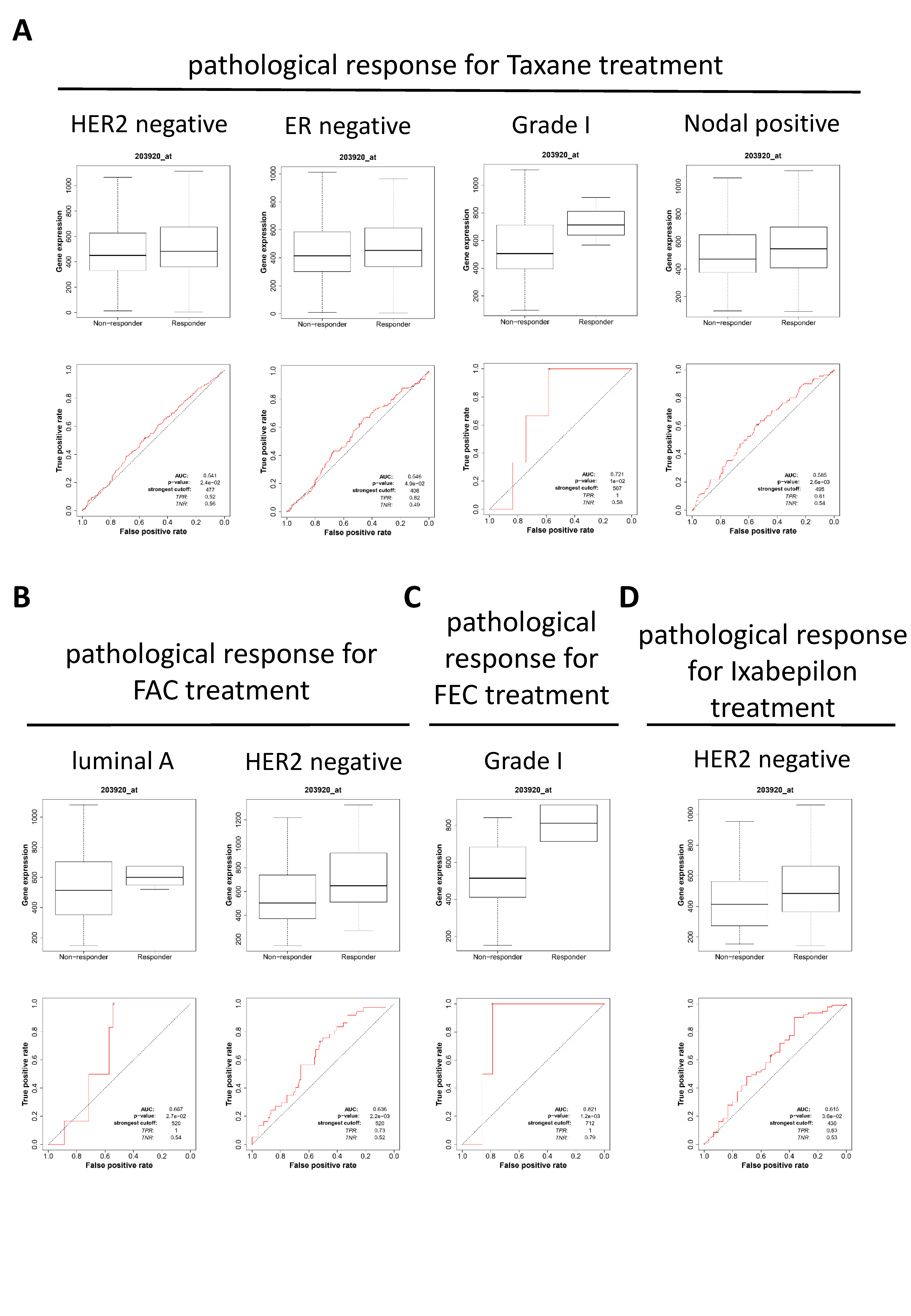

Supplement: Supplementary file 7 [file Image15.TIFF]

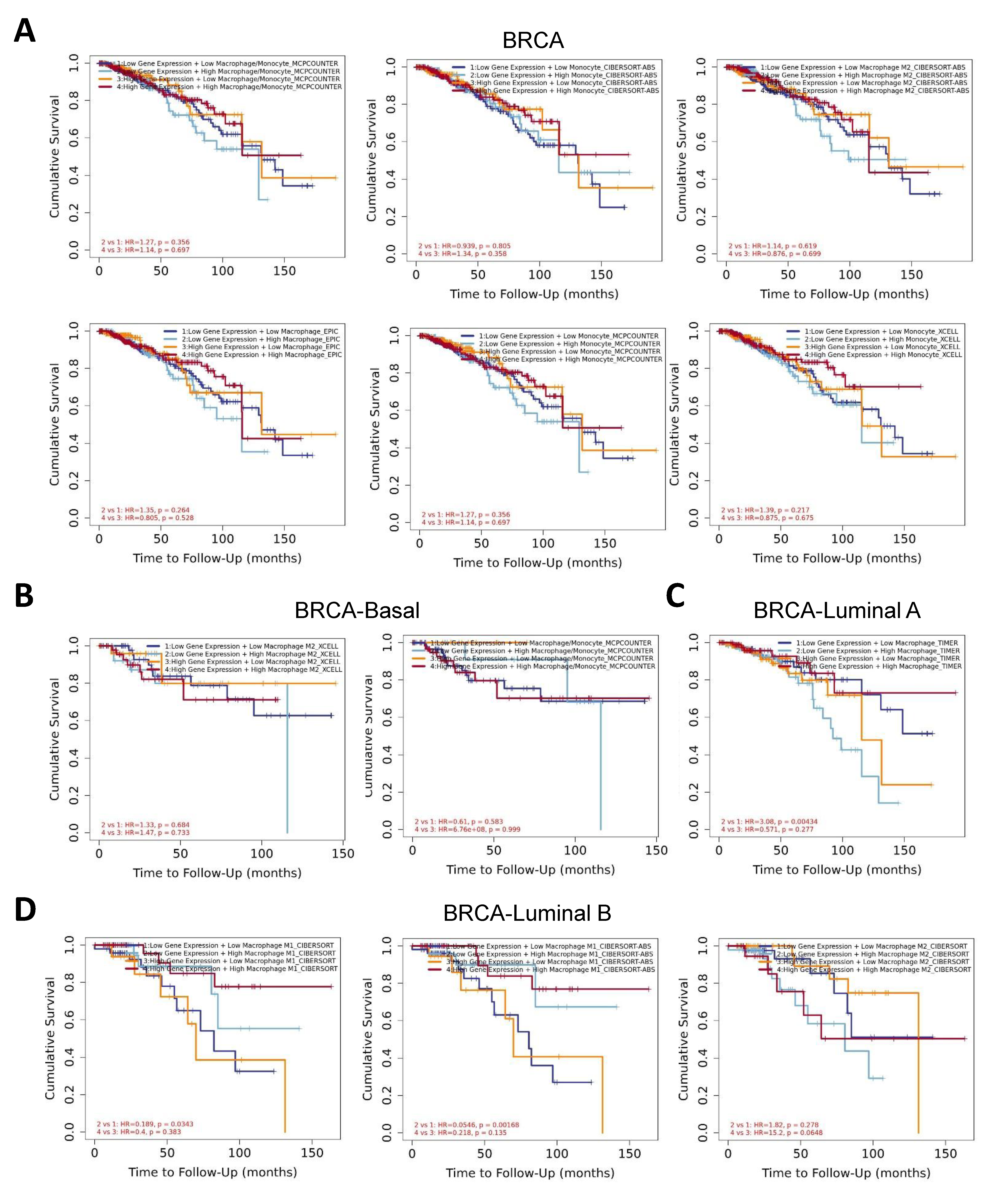

Supplement: Supplementary file 8 [file Image8.TIFF]

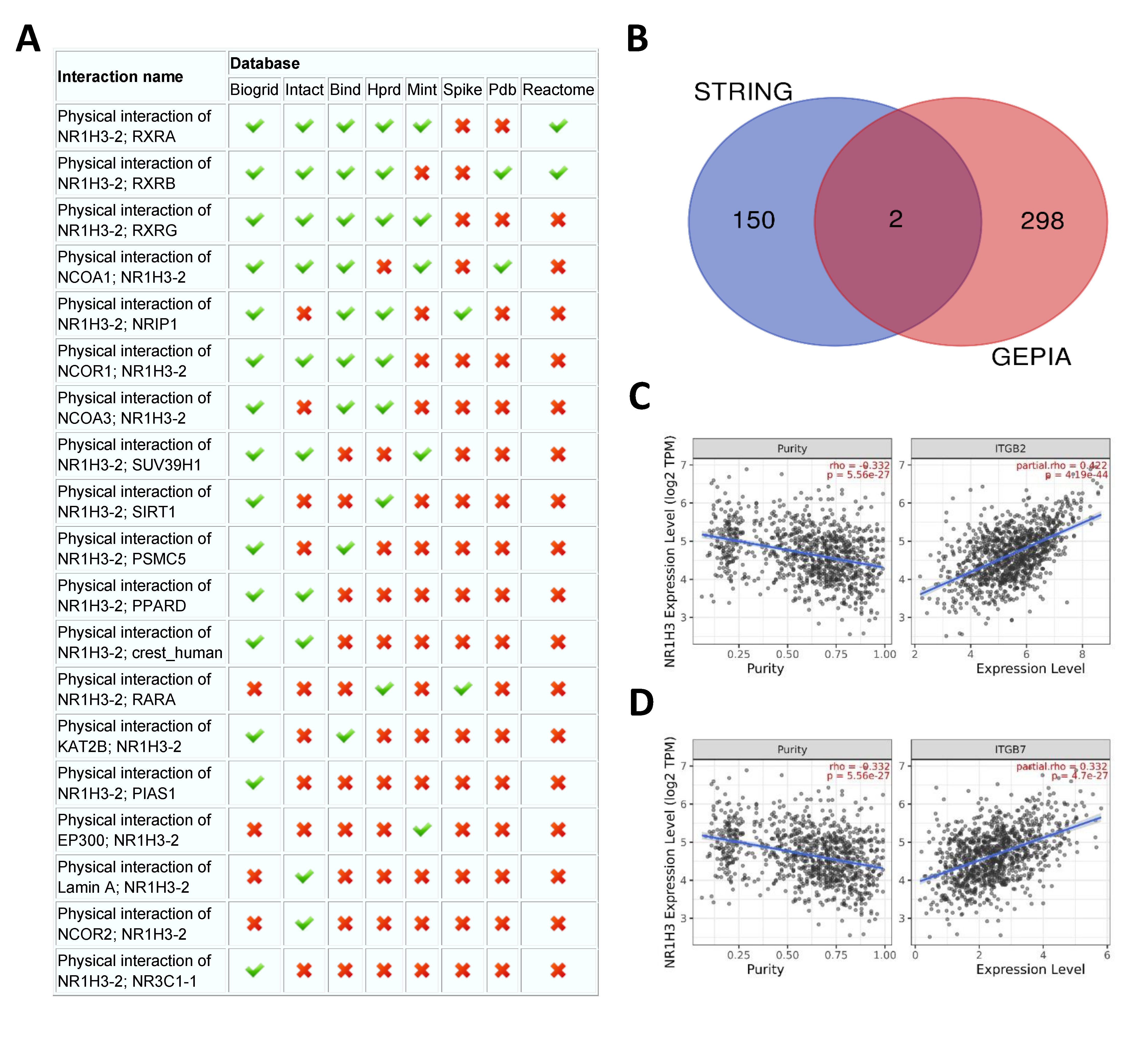

Supplement: Supplementary file 9 [file Image11.TIFF]

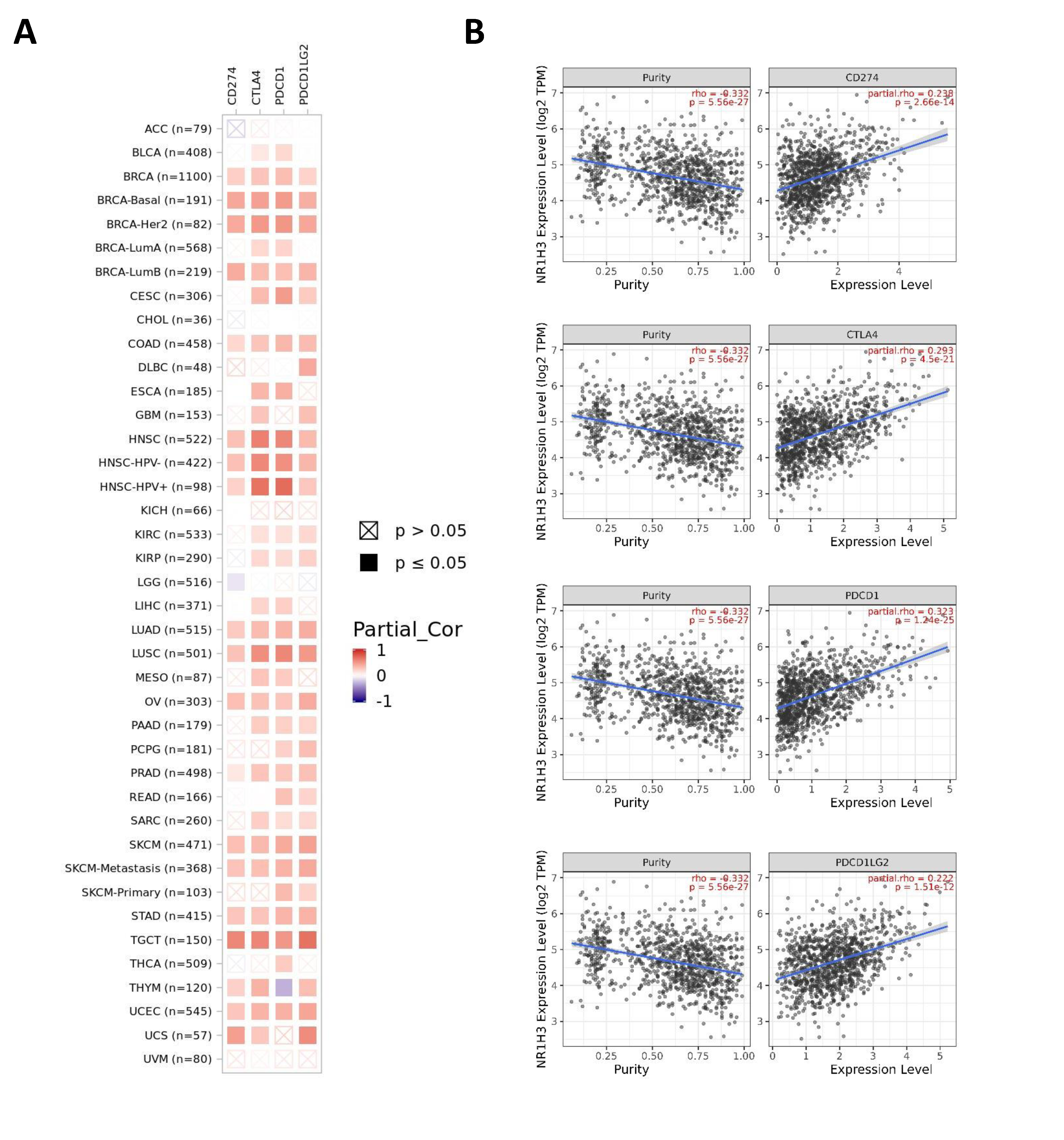

Supplement: Supplementary file 10 [file Image10.TIFF]

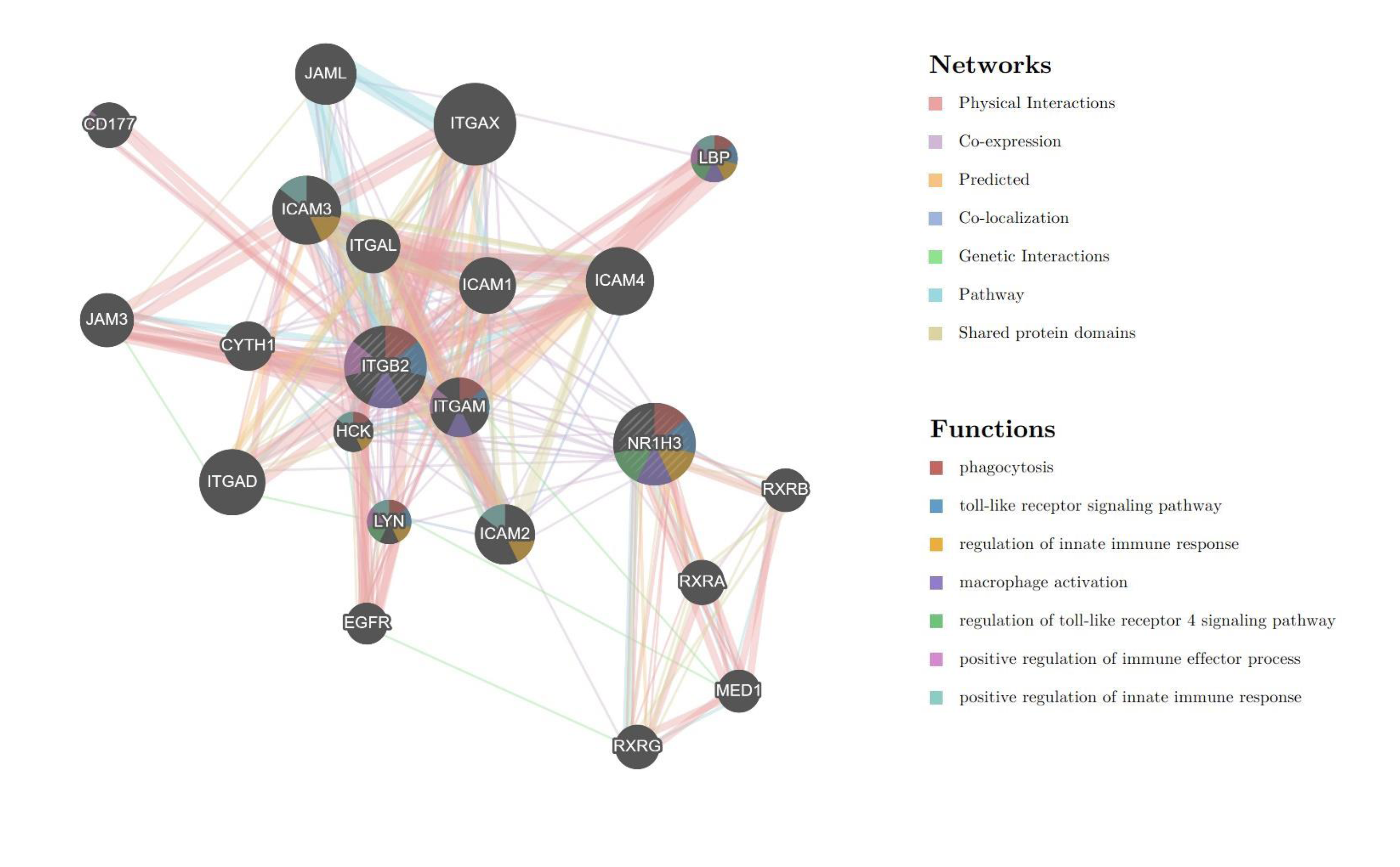

Supplement: Supplementary file 11 [file Image12.TIFF]

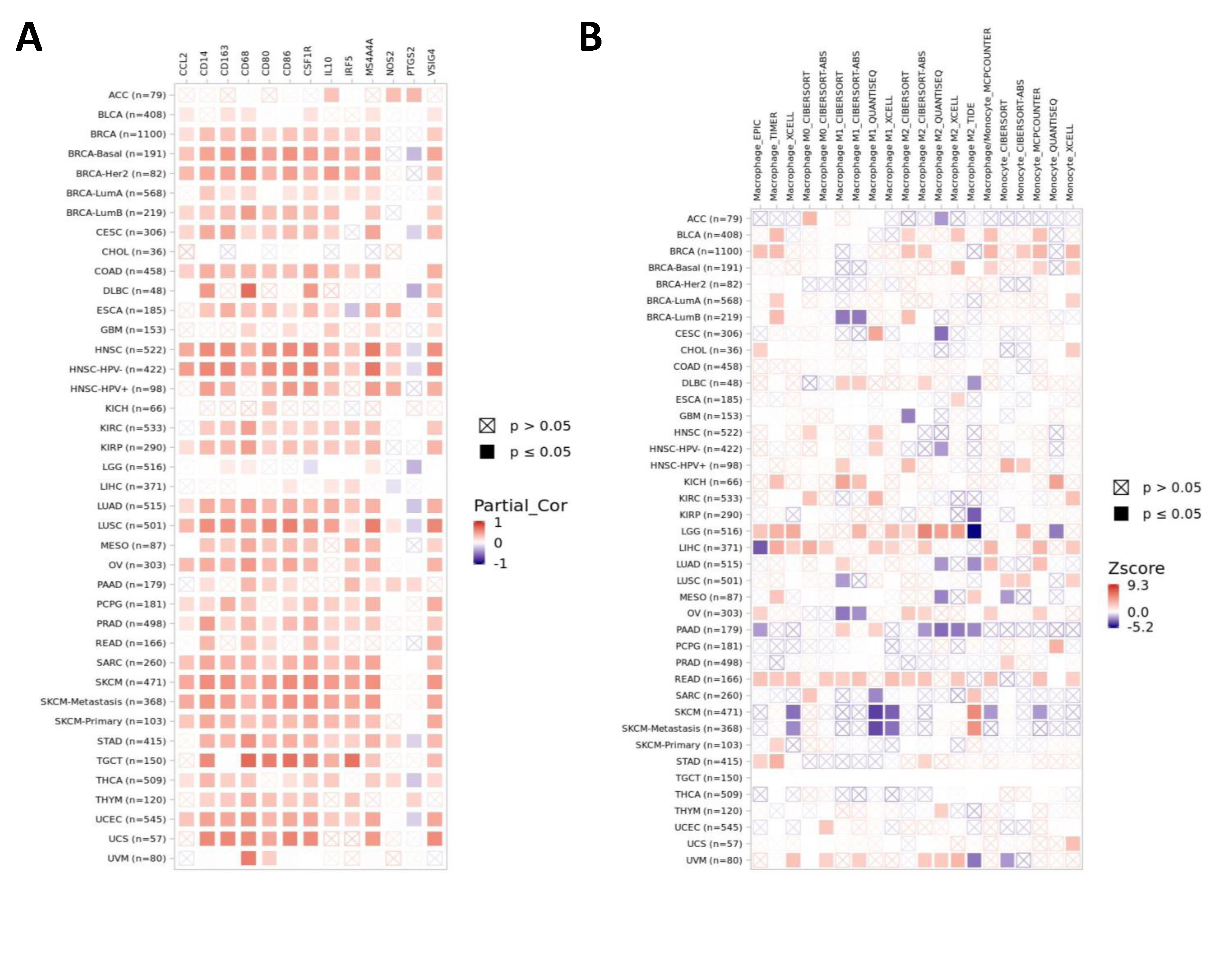

Supplement: Supplementary file 12 [file Image6.TIFF]

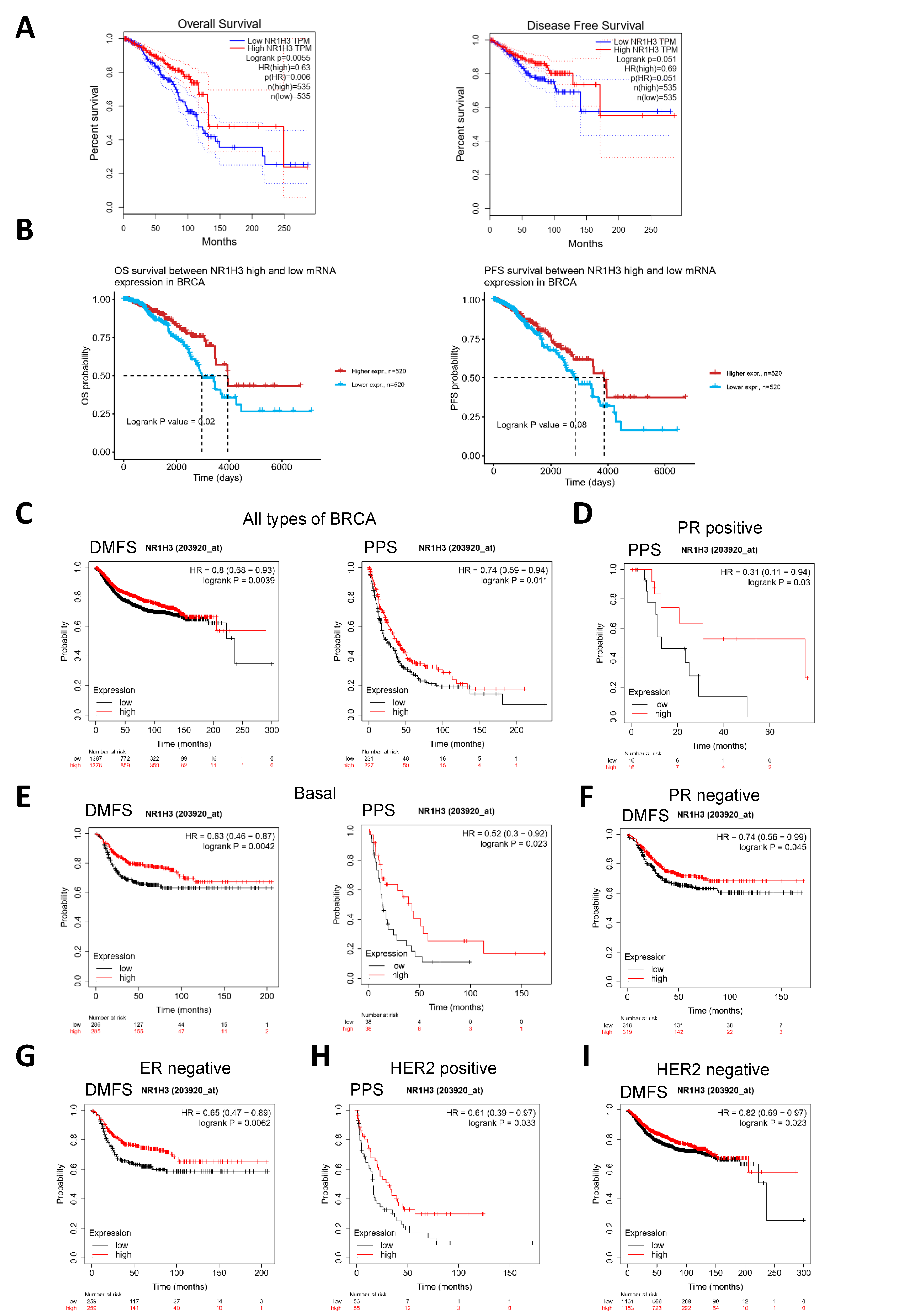

Supplement: Supplementary file 13 [file Image2.TIFF]

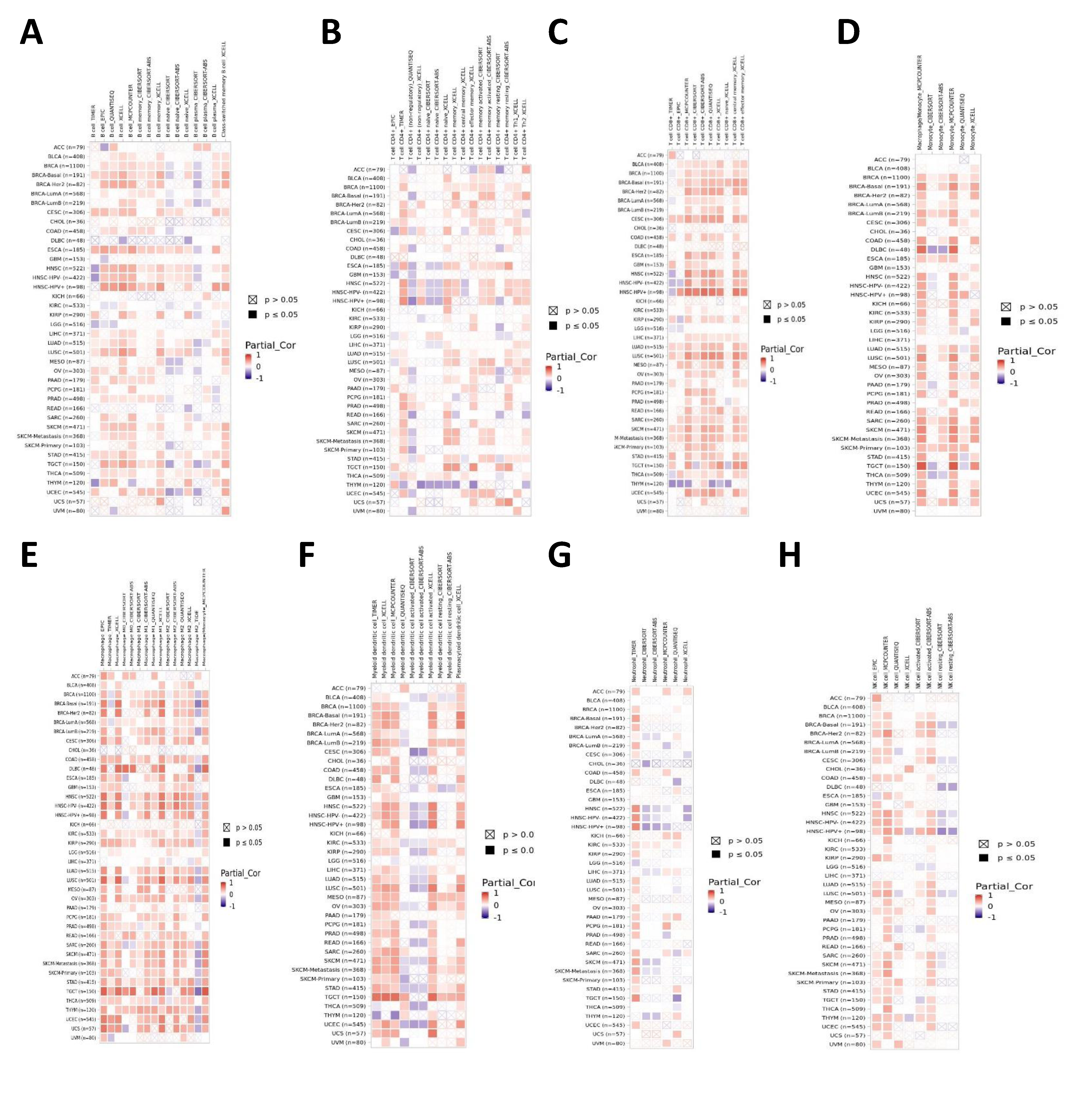

Supplement: Supplementary file 14 [file Image4.TIFF]

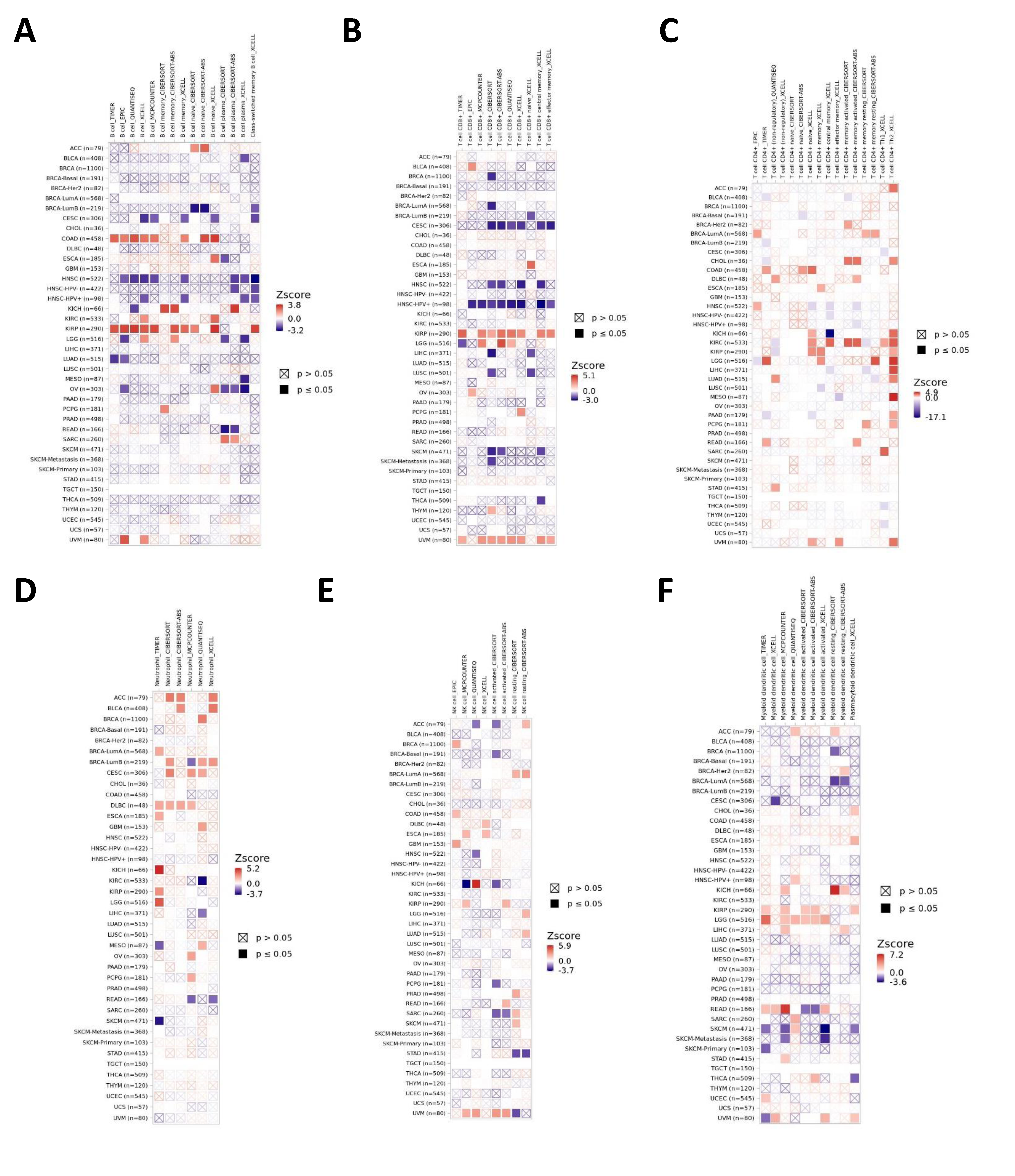

Supplement: Supplementary file 15 [file Image7.TIFF]
